# Bioinformatics Identification of Anti-CRISPR Loci by Using Homology, Guilt-by-Association, and CRISPR Self-Targeting Spacer Approaches

**DOI:** 10.1128/mSystems.00455-19

**Published:** 2019-09-10

**Authors:** Yanbin Yin, Bowen Yang, Sarah Entwistle

**Affiliations:** a Nebraska Food for Health Center, Department of Food Science and Technology, University of Nebraska—Lincoln, Lincoln, Nebraska, USA; b Department of Biological Sciences, Northern Illinois University, DeKalb, Illinois, USA; University College Cork

**Keywords:** Aca, Acr, CRISPR self-targeting, CRISPR-Cas, anti-CRISPR, gene neighborhood, genomic island, helix-turn-helix, phage, prophage

## Abstract

Anti-CRISPR (Acr) loci/operons encode Acr proteins and Acr-associated (Aca) proteins. Forty-five Acr families have been experimentally characterized inhibiting seven subtypes of CRISPR-Cas systems. We have developed a bioinformatics pipeline to identify genomic loci containing Acr homologs and/or Aca homologs by combining three computational approaches: homology, guilt-by-association, and self-targeting spacers. Homology search found thousands of Acr homologs in bacterial and viral genomes, but most are homologous to AcrIIA7 and AcrIIA9. Investigating the gene neighborhood of these Acr homologs revealed that only a small percentage (23.0% in bacteria and 8.2% in viruses) of them have neighboring Aca homologs and thus form Acr-Aca operons. Surprisingly, although a self-targeting spacer is a strong indicator of the presence of Acr genes in a genome, a large percentage of Acr-Aca loci are found in bacterial genomes without self-targeting spacers or even without complete CRISPR-Cas systems. Additionally, for Acr homologs from genomes with self-targeting spacers, homology-based Acr family assignments do not always agree with the self-targeting CRISPR-Cas subtypes. Last, by investigating Acr genomic loci coexisting with self-targeting spacers in the same genomes, five known subtypes (I-C, I-E, I-F, II-A, and II-C) and five new subtypes (I-B, III-A, III-B, IV-A, and V-U4) of Acrs were inferred. Based on these findings, we conclude that the discovery of new anti-CRISPRs should not be restricted to genomes with self-targeting spacers and loci with Acr homologs. The evolutionary arms race of CRISPR-Cas systems and anti-CRISPR systems may have driven the adaptive and rapid gain and loss of these elements in closely related genomes.

**IMPORTANCE** As a naturally occurring adaptive immune system, CRISPR-Cas (clustered regularly interspersed short palindromic repeats–CRISPR-associated genes) systems are widely found in bacteria and archaea to defend against viruses. Since 2013, the application of various bacterial CRISPR-Cas systems has become very popular due to their development into targeted and programmable genome engineering tools with the ability to edit almost any genome. As the natural off-switch of CRISPR-Cas systems, anti-CRISPRs have a great potential to serve as regulators of CRISPR-Cas tools and enable safer and more controllable genome editing. This study will help understand the relative usefulness of the three bioinformatics approaches for new Acr discovery, as well as guide the future development of new bioinformatics tools to facilitate anti-CRISPR research. The thousands of Acr homologs and hundreds of new anti-CRISPR loci identified in this study will be a valuable data resource for genome engineers to search for new CRISPR-Cas regulators.

## Abstract (precis)

As a naturally occurring adaptive immune system, CRISPR-Cas (clustered regularly interspersed short palindromic repeats–CRISPR-associated genes) systems are widely found in bacteria and archaea to defend against viruses. Since 2013, the application of various bacterial CRISPR-Cas systems has become very popular due to their development into targeted and programmable genome engineering tools with the ability to edit almost any genome. As the natural off-switch of CRISPR-Cas systems, anti-CRISPRs have a great potential to serve as regulators of CRISPR-Cas tools and enable safer and more controllable genome editing. This study will help understand the relative usefulness of the three bioinformatics approaches for new Acr discovery, as well as guide the future development of new bioinformatics tools to facilitate anti-CRISPR research. The thousands of Acr homologs and hundreds of new anti-CRISPR loci identified in this study will be a valuable data resource for genome engineers to search for new CRISPR-Cas regulators.

## INTRODUCTION

CRISPR-Cas (clustered regularly interspersed short palindromic repeats–CRISPR-associated genes) is an antivirus mechanism found in ∼40% of sequenced bacterial genomes and ∼80% of archaeal genomes (1). As prokaryotes and viruses have battled for billions of years, it is not surprising that viruses have evolved various ways to escape the CRISPR-Cas attack (2, 3). One newly discovered anti-CRISPR-Cas mechanism is viruses encoding small proteins to directly interact with Cas enzymes to prevent the destruction by the CRISPR-Cas systems (4, 5).

The first such small viral protein, called anti-CRISPR (Acr) protein, was discovered in 2013 in *Pseudomonas* phages and prophages (6). Acr-encoding genes often have an immediate downstream gene coding for a putative transcription regulator named Aca (Acr-associated) protein (7, 8), although exceptions have been recently reported in lytic phages and certain Acr types (9–12). Acr-Aca loci (or operons) have since been experimentally characterized in phages and prophages of different bacterial species, as well as an archaeal virus, Sulfolobus islandicus rudivirus 3 (SIRV3) (13), which have been summarized in recent review papers (4, 7, 8, 14, 15).

Sequence comparison found that published Acr proteins are very divergent in sequence, and most of them do not have known functional domains in the database (e.g., Pfam [16]). As of July 2019, the experimentally characterized Acrs form 45 sequence similarity-based families (representative proteins in http://bcb.unl.edu/AcrDB/Download/knownAcrAca/known-loci.xlsx and https://tinyurl.com/anti-CRISPR [17]). In contrast, Aca is more conserved, and seven distantly related Aca families have been reported (11), all having a helix-turn-helix (HTH) DNA binding domain, hypothetically regulating the expression of Acr genes.

Bioinformatics sequence analysis has been critical in the characterization of the previous 45 Acr families (14). The widely accepted sequence homology search is deemed a very accurate bioinformatics approach and has found hundreds of homologs of known Acr proteins (11, 12, 18, 19). However, the sequence similarity search alone has limited use for identifying new Acr families targeting new CRISPR-Cas subtypes. Instead, searching for HTH domains in the more conserved Aca proteins and then using gene neighborhood to probe new Acrs has proven to be very successful, known as the guilt-by-association (GBA) approach (20). Additionally, another approach using the self-targeting idea, i.e., bacterial genomes having CRISPR spacers and their targets (i.e., protospacers) coexisting in the same genome, has also been applied to searching for new Acrs (11, 12, 21). Our goal in this paper was to perform large-scale and comprehensive bioinformatics data mining for Acr-Aca loci in all sequenced bacterial genomes by combining sequence homology, GBA, and self-targeting approaches.

Specifically, by a systematic analysis of thousands of Acr homologs and their gene neighborhoods, we hoped to address the following unknown questions. (i) How do known Acr families differ in terms of sequence conservation and taxonomic and environmental distribution, as well as genomic context? (ii) Do Acr homologs tend to coexist with Aca homologs in the same operon? (iii) Do Acr homologs tend to be found in genomes with self-targeting spacers and CRISPR-Cas systems? (iv) For genomes with both Acr homologs and self-targeting spacers, do homology-based Acr family assignments agree with the self-targeting CRISPR-Cas subtypes? Answers to these questions will significantly improve our understanding of the genomic properties and adaptive evolution of anti-CRISPR loci.

Furthermore, according to the most recent research, there are two classes of CRISPR-Cas systems in prokaryotes further classified into six types including at least 25 subtypes (22). The 45 experimentally characterized Acr families inhibit only seven subtypes (I-C, I-D, I-E, I-F, II-A, II-C, and V-A) of three CRISPR-Cas types, among which I-C and I-D have only a single characterized Acr protein. It was speculated that most CRISPR-Cas subtypes should have a corresponding anti-CRISPR systems (4, 14). Therefore, we also aimed to offer some evidence for this hypothesis by a systematic search and mapping of CRISPR-Cas and anti-CRISPR loci identified in the surveyed genomes.

## RESULTS AND DISCUSSION

**FIG 1:**
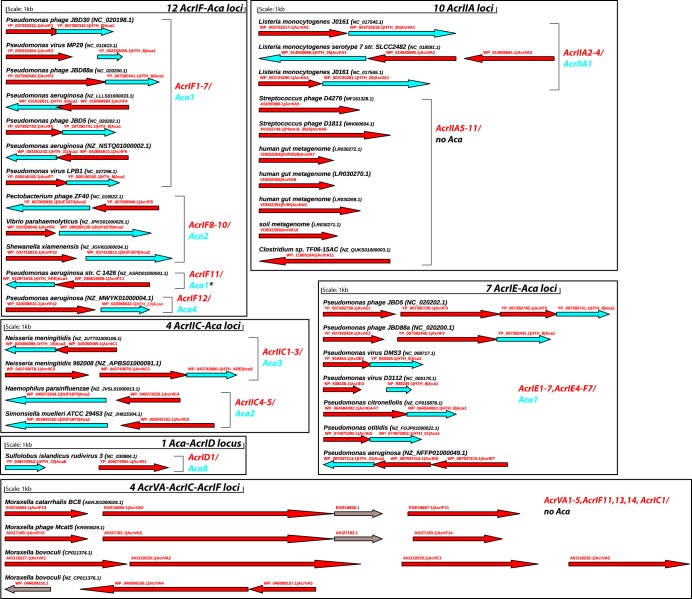
Genomic context of 45 experimentally characterized Acr representative genes. In total, 38 genomic loci are shown. Each locus contains at least one Acr gene (red arrows). There are 26 loci that also contain Aca genes (cyan arrows) with Pfam HTH or DUF1870 domains. Seven AcrIIA genes do not have neighboring Aca genes. The five AcrVA genes form four loci that also contain AcrIF genes, an AcrIC gene, and functional unknown genes (gray arrows) but no Aca genes. The plots were made with the Gene Graphics server (38). *, AcrF11 homologs were found to be next to other HTH domain-containing proteins as well, which were named Aca4 through Aca7 in reference 11. The archaeal viral AcrID1 has an upstream HTH protein, which has never been officially named and is named Aca8 here.

The sequence features of the 45 published Acr proteins and associated Aca proteins are detailed in Text S1 in the supplemental material. These sequence features were critical for the computational identification of new Acr families. In brief, the 45 Acr families include 14 AcrIF families (11, 20), seven AcrIE families (11, 23), one AcrIE/IF hybrid family (11), one AcrIC family (11), one AcrID family (13), 11 AcrIIA families (9, 10, 21, 24, 25), five AcrIIC families (26, 27), and five AcrVA families (11, 12) (https://tinyurl.com/anti-CRISPR). Representative genes of the 45 Acr families and their genomic context are graphically presented in Fig. 1. These genes form in total 38 genomic operons, of which 26 contain Aca genes and thus are named Acr-Aca loci.

10.1128/mSystems.00455-19.1 TEXT S1 Supplementary results. Download Text S1, DOCX file, 0.3 MB. Copyright © 2019 Yin et al. 2019 Yin et al. This content is distributed under the terms of the Creative Commons Attribution 4.0 International license.

### Bioinformatics pipeline for Acr-Aca locus identification.

**FIG 2:**
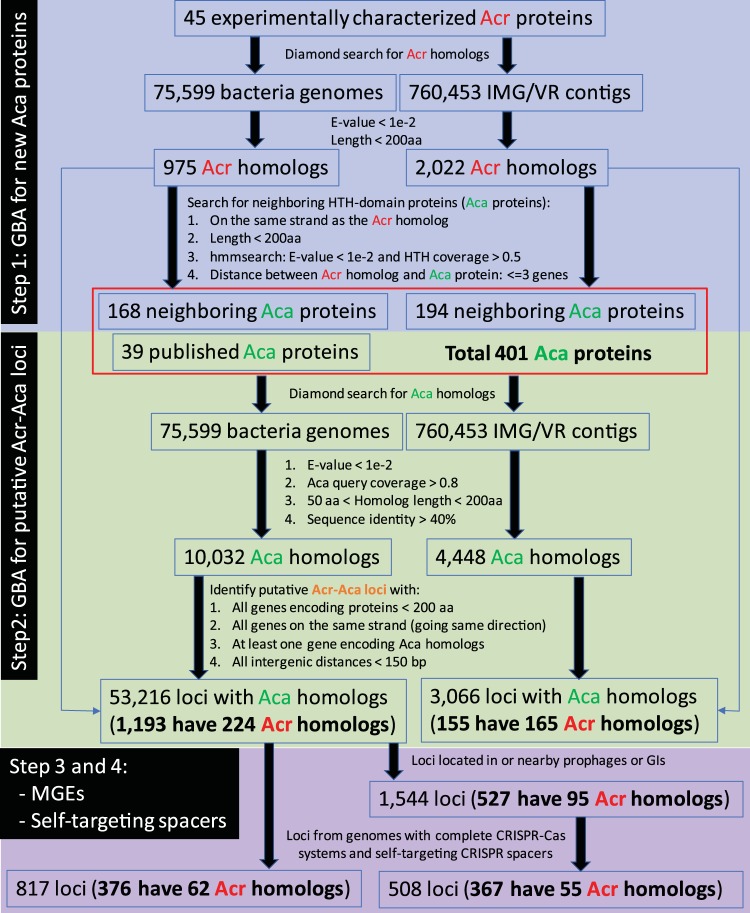
Bioinformatics pipeline to identify genomic loci containing Acr homologs and/or Aca homologs. Guilt-by-association (GBA) was used to identify new HTH proteins (Aca proteins) that neighbor homologs of known Acr proteins in the RefSeq bacterial genomes and IMG/VR metagenome-assembled viral contigs (MACs). These Aca proteins were combined with published Aca proteins (Text S1) to search for more Aca homologs. GBA was further used to identify genomic loci (or operons) that contain Aca homologs. More filters were then used to select loci that are from genomes with annotated mobile genetic elements (MGEs), complete CRISPR-Cas systems, and self-targeting CRISPR spacers. For Diamond search (39), the *–more-sensitive* option was used to improve search sensitivity. For hmmsearch (http://hmmer.org/), the default parameters were used.

As illustrated in Fig. 2, a bioinformatics pipeline was developed with four steps of data processing. This pipeline can find all the 26 published Acr-Aca loci depicted in Fig. 1 and therefore has a recall of 100%.

In the first step, we aimed to identify homologs of known Acr proteins and then use GBA (gene neighborhood) to identify new Aca proteins, whose encoding genes are located in proximity to Acr homologs (see Materials and Methods). In total, 975 unique RefSeq proteins (one protein identifier [ID] can be found in multiple very similar RefSeq bacterial strain/isolate genomes) and 2,022 IMG/VR (28) proteins were found to be Acr homologs. Searching for HTH domain-containing proteins surrounding these Acr homologs found 168 and 194 Aca proteins (Fig. 2 gives criteria) in RefSeq and IMG/VR, respectively.

In the second step, 401 Aca proteins (see Materials and Methods) were used as query for homology search, and as a result, 10,032 and 4,448 highly confident Aca homologs of the two databases (Fig. 2 gives criteria), respectively, were kept for further identifying putative Acr-Aca loci. After this step, we found 53,216 loci (first column of data in Table 1) meeting the required criteria (see Materials and Methods). Interestingly, 1,193 (2.2%) of these loci also contain 224 unique Acr homologs (out of the 975 Acr homologs of step 1). Similarly, we found 3,066 genomic loci from 3,013 viral/proviral contigs of the IMG/VR database, which include 155 loci containing 165 Acr homologs (out of the 2,022 Acr homologs of step 1).

**TABLE 1:** Genomic loci with Aca homologs and with Acr plus Aca homologs

Parameter	Value by filter steps in Fig. 2			
	1, 2	1, 2, 3	1, 2, 3, 4	1, 2, 4
No. of loci with Aca homologs	53,216	1,544	508	817
No. of genomes	29,365	1,310	478	672
No. (%) of genomes with >1 locus	14,405 (49.1)	184 (14.0)	20 (4.2)	97 (14.4)
No. (%) of genomes with self-targeting spacers	672 (2.3)	478 (36.5)	478 (100)	672 (100)

No. (%) of loci with Aca + Acr homologs	1,193 (2.2)	527 (34.1)	367 (72.2)	376 (46.0)
No. (%) of genomes	1,102 (3.8)	511 (39.0)	363 (75.9)	370 (55.1)
No. (%) of genomes with >1 locus	81 (7.4)	13 (2.5)	4 (1.1)	6 (1.6)
No. (%) of genomes with self-targeting spacers	370 (33.6)	363 (71.0)	363 (100)	370 (100)
No. (%) of Acr homologs (unique IDs)	224 (100)	95 (42.4)	55 (24.6)	62 (27.7)
No. (%) of Aca homologs (unique IDs)	157 (100)	66 (42.0)	39 (24.8)	45 (28.7)

In the third step, we kept only RefSeq loci located within or near annotated prophages and genomic islands (GIs) according to two public mobile genetic element (MGE) databases (see Materials and Methods). After this step, only 1,544 genomic loci from 1,310 bacterial genomes remained (second column of data in Table 1). This substantial decrease is likely because most RefSeq genomes of this study were not covered in the two MGE databases (29, 30). Among these 1,544 loci, 527 also contain 95 unique Acr homologs. The 3,066 loci from the IMG/VR database were not analyzed further, because they are from viral/proviral contigs that are MGEs by themselves.

In the fourth step, we further filtered the 1,544 loci to keep only those having self-targeting CRISPR spacers based on the data from Watters et al. (12). Specifically, we required that the bacterial genome of each Acr-Aca locus must have a complete CRISPR-Cas system (including a complete set of Cas enzymes and a complete nearby CRISPR array) and at least one CRISPR spacer targeting a protospacer located in the same genomic contig or chromosome as the Acr-Aca locus (see Materials and Methods). With these stringent criteria, 508 genomic loci remained (third column of data in Table 1), and a majority of them (367 or 72.2%) contain 55 unique Acr homologs.

Additionally, when the third step was skipped, 817 genomic loci were found in 672 genomes (fourth column of data in Table 1), which passed filters 1, 2, and 4. Among these loci, 376 contain both Aca and Acr homologs. The confidence level of the different genomic locus data sets (Table 1) generated from this pipeline was discussed in Text S1 in the supplemental material, particularly in the context of the usefulness of the three approaches (homology, GBA, and self-targeting spacer) for new Acr-Aca locus identification.

### Thousands of Acr homologs are found in bacterial and viral genomes, but most are homologous to AcrIIA7 and AcrIIA9.

**FIG 3:**
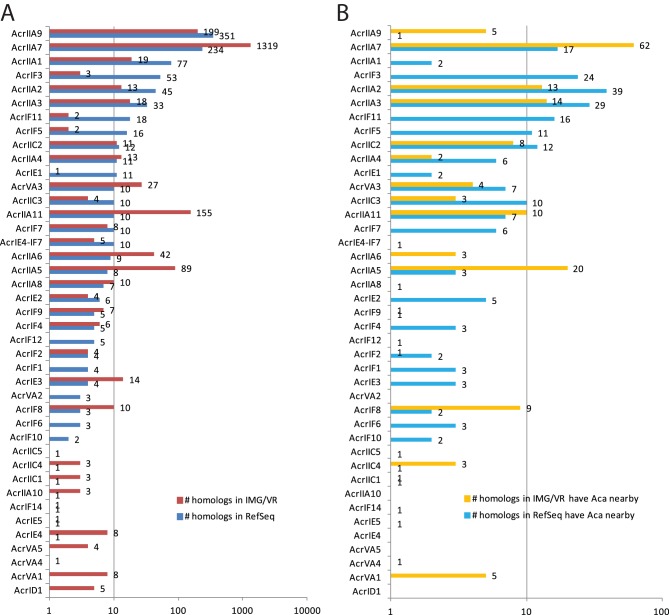
Homologs of known Acr families in RefSeq and IMG/VR databases. (A) Breakdown numbers of homologs in each known Acr family (total of 975 for RefSeq and 2,022 for IMG/VR). (B) Breakdown numbers of homologs in each known Acr family that have neighboring Aca homologs (total of 224 for RefSeq and 165 for IMG/VR [Fig. 2]). Note that AcrIIA1 itself is considered an Aca; therefore, the two RefSeq AcrIIA1 genes in panel B coexist with other AcrIIA1 homologs in the same operons.

We have focused on the 975 RefSeq and 2,022 IMG/VR Acr homologs that were identified by homology search. Figure 3A and Table S1 show that 37 out of the 45 known Acr families have homologs in the RefSeq bacterial genomes (blue bars in Fig. 3A) and 35 have homologs in the IMG/VR viral genomes (red bars in Fig. 3A). In agreement with references 24 and 25, AcrIIA7, AcrIIA9, and AcrIIA11 are among the families with the most homologs, particularly in IMG/VR. AcrIIA7 and AcrIIA9 together have 585 homologs in RefSeq and 1,516 homologs in IMG/VR, which account for 60.0% of the 975 RefSeq homologs and 75.1% of the 2,022 IMG/VR homologs. Note that AcrIIA7 has the Pfam DUF2829 domain and AcrIIA9 has the Pfam PcfK domain, which make them more conserved Acr families.

10.1128/mSystems.00455-19.2 TABLE S1 Acr homolog counts in RefSeq and IMG/VR databases. Download Table S1, XLSX file, 0.02 MB. Copyright © 2019 Yin et al. 2019 Yin et al. This content is distributed under the terms of the Creative Commons Attribution 4.0 International license.

In terms of the taxonomic distribution (Table S2), AcrIIA7 homologs were found in six phyla, followed by AcrIIA9 homologs found in three phyla. Similar findings have been made in reference 24. Homologs of AcrIIA11, AcrVA2, and AcrVA3 were found in two phyla. The rest of the families were found in only one phylum. In fact, 28 (75.7%) Acr families have homologs restricted to one class, and 22 (59.5%) have homologs restricted to one genus, which makes them qualify for the definition of orphan genes (31, 32). For the habitat distribution (Table S3), AcrIIA7, AcrIIA9, and AcrIIA11 were the most widely distributed, in line with references 24 and 25. AcrIIA7 and AcrIIA9 homologs were mostly found in host-associated habitat (e.g., human gut), while the top habitats for AcrIIA11 homologs were waters.

10.1128/mSystems.00455-19.3 TABLE S2 Taxonomic distribution of 975 Acr homologs in RefSeq. Download Table S2, XLSX file, 0.01 MB. Copyright © 2019 Yin et al. 2019 Yin et al. This content is distributed under the terms of the Creative Commons Attribution 4.0 International license.

10.1128/mSystems.00455-19.4 TABLE S3 Habitat distribution of 2,022 Acr homologs in IMG/VR. Download Table S3, XLSX file, 0.01 MB. Copyright © 2019 Yin et al. 2019 Yin et al. This content is distributed under the terms of the Creative Commons Attribution 4.0 International license.

With respect to genomic context, the 975 RefSeq homologs are located in 4,462 genomic loci (all genes on the same strand encoding proteins of <200 amino acids [aa]) encoding in total 3,577 unique protein IDs. Searching the 2,602 non-Acr proteins against the Pfam database found that 759 (29.2%) proteins can be functionally annotated and at least 142 (18.7%) have HTH domains, and 161 (21.2%) have phage-related domains (Table S4). In addition, domains of unknown function (DUF) and other bacterial defense-related domains are also prevalent. For example, DUF1896 (hypothetical proteins mainly present in *Bacteroidetes*) was found in 98 proteins surrounding AcrIIA9 homologs, ArdA (proteins targeting restriction-modification system of bacteria) was found in 56 proteins surrounding AcrIIA9 homologs, DUF2829 (hypothetical proteins mainly present in *Firmicutes* and *Proteobacteria*) was found in 23 proteins surrounding AcrIIA7 homologs, and various toxin-antitoxin domains (e.g., ParE_toxin, YdaS_antitoxin, and HigB-like_toxin, related to programmed cell death) were found in at least 12 proteins surrounding different AcrIIA and AcrIF proteins.

10.1128/mSystems.00455-19.5 TABLE S4 Pfam domain analysis of proteins surrounding the 975 Acr homologs in RefSeq. Download Table S4, XLSX file, 0.01 MB. Copyright © 2019 Yin et al. 2019 Yin et al. This content is distributed under the terms of the Creative Commons Attribution 4.0 International license.

Similarly, the 2,022 IMG/VR homologs were located in 1,980 genomic loci encoding in total 10,500 proteins. Searching the 8,478 non-Acr proteins against the Pfam database found that 1,425 (16.8%) can be functionally annotated (Table S5). Although all of these proteins are from viruses/proviruses, only 137 (9.6%) contain HTH domains.

10.1128/mSystems.00455-19.6 TABLE S5 Pfam domain analysis of proteins surrounding the 2,022 Acr homologs in IMG/VR. Download Table S5, XLSX file, 0.01 MB. Copyright © 2019 Yin et al. 2019 Yin et al. This content is distributed under the terms of the Creative Commons Attribution 4.0 International license.

These results and the following gene neighborhood results significantly expanded the published analyses (24, 25), which typically focused on AcrIIA families and did not look at the genomic context of Acr homologs and the presence of self-targeting spacers in the genomes.

### Only a small percentage (23.0% in bacteria and 8.2% in viruses) of Acr homologs have neighboring Aca homologs and form Acr-Aca operons.

As shown in Fig. 2 and Table 1, only 23.0% RefSeq (224 out of 975) and 8.2% IMG/VR (165 out of 2,022) homologs have surrounding Aca homologs, which form the highly confident 1,193 (Data Set S1) and 155 (Data Set S2) Aca-Acr loci in the two databases. Genomic loci without recognized Aca genes may encode unknown Aca proteins with non-HTH domains (see Pfam domain analysis of non-Acrs above) or encode Acr proteins that can function alone without the help of Acas (such as AcrVA genes and AcrIIA5 to AcrIIA11 genes shown in Fig. 1). The much lower percentage (8.2%) in IMG/VR indicates that Acrs in viral genomes are more likely to work without Acas.

10.1128/mSystems.00455-19.8 DATA SET S1 One thousand one hundred ninety-three RefSeq genomic loci with both Acr and Aca homologs. Download Data Set S1, XLS file, 1.7 MB. Copyright © 2019 Yin et al. 2019 Yin et al. This content is distributed under the terms of the Creative Commons Attribution 4.0 International license.

10.1128/mSystems.00455-19.9 DATA SET S2 One hundred fifty-five IMG/VR genomic loci with both Acr and Aca homologs. Download Data Set S2, XLS file, 0.3 MB. Copyright © 2019 Yin et al. 2019 Yin et al. This content is distributed under the terms of the Creative Commons Attribution 4.0 International license.

Further analysis of the 224 Acr homologs (Fig. 3B, light blue bars) found that, while most AcrIE, AcrIF, and AcrIIC families have over 50% of RefSeq homologs with a neighboring Aca homolog, others do not (Table S1). Specifically, only one (0.3%) of the 351 AcrIIA9 homologs and 17 (7.3%) of the 234 AcrIIA7 homologs have neighboring Aca homologs (Fig. 3B). Some other Acr families also have very low percentages of homologs with surrounding Acas in RefSeq, e.g., AcrIIA6 (0%), AcrIIA8 (14.3%), AcrVA2 (0%), AcrIE1 (18.2%), AcrIE4-IF7 (10.0%), AcrIF12 (20.0%), and AcrIF9 (20.0%) (Table S1).

Interestingly, of the 12 published Acrs (AcrIIA5 to AcrIIA11 and AcrVA1 to AcrVA5) without surrounding Acas (Fig. 1), nine have at least one homolog with neighboring Aca homologs in either RefSeq or IMG/VR (Fig. 3B and Table S1). For example, AcrVA1 has eight homologs in IMG/VR viral genomes (Fig. 3A), and five of them (Fig. 3B) are located next to a protein with HTH_17 or HTH_XRE domains (Table S5). This is reminiscent of the previous finding (11) that AcrIF11 homologs can be next to different Aca genes or next to no Acas but AcrVA genes (Fig. 1). All these suggest the adaptive evolution of the Acr gene neighborhood likely through frequent recombinations and gene transfers.

In terms of the taxonomic distribution of the 224 Acr homologs (Table S6), the 17 AcrIIA7 homologs are found in three phyla (*Proteobacteria*, *Firmicutes*, and *Cyanobacteria*), the seven AcrVA3 homologs are found in two (*Firmicutes* and *Proteobacteria*), and the rest of the families are found in only one phylum. A further examination of the species origin found that the 224 Acr homologs of the 1,193 Acr-Aca loci are from 1,102 genomes of only 62 species, and 81.4% of these genomes are from two species: Pseudomonas aeruginosa (647 genomes) and Listeria monocytogenes (250 genomes). We believe that this highly biased species distribution is partially because most known AcrIE, -IF, and -IIA proteins and their associated Acas were from the two species (Fig. 1), and thus, the homology search using these unconserved query proteins resulted in hits restricted to these species.

10.1128/mSystems.00455-19.7 TABLE S6 Taxonomic distribution of 224 Acr homologs in RefSeq that have neighboring Aca homologs. Download Table S6, XLSX file, 0.01 MB. Copyright © 2019 Yin et al. 2019 Yin et al. This content is distributed under the terms of the Creative Commons Attribution 4.0 International license.

### A large percentage of Acr-Aca loci are found in bacterial genomes without self-targeting spacers or even without complete CRISPR-Cas systems.

Of the 26 published Acr-Aca loci shown in Fig. 1, 16 are from bacteria. Surprisingly, only four (25.0%) of the 16 loci have complete CRISPR-Cas systems and self-targeting CRISPR spacers according to the work of Watters et al. (12). Using CRISPRCasFinder (33) and our in-house programs, we found that five additional loci are from genomes with CRISPR-Cas systems but no self-targeting spacers. The remaining seven loci are from genomes without even a complete CRISPR-Cas system (Table 2).

**TABLE 2:** Sixteen experimentally characterized Acr-Aca loci in bacterial genomes

Bacterial species	RefSeq genome ID	Published Acr-Aca loci	Presence of complete CRISPR-Cas and self-targeting spacer
Listeria monocytogenes J0161	GCF_000168635	AcrIIA2-AcrIIA1	Yes
Listeria monocytogenes serotype 7 strain SLCC2482	GCF_000210795	AcrIIA2-AcrIIA3-AcrIIA1	Yes
Listeria monocytogenes J0161	GCF_000168635	AcrIIA4-AcrIIA1	Yes
Pseudomonas otitidis	GCF_900111835	AcrIE5-Aca1	Yes
Pseudomonas aeruginosa WH-SGI-V-07059	GCF_001450485^*a*^	AcrIF4-AcrIE3-Aca1	No self-target spacer
Haemophilus parainfluenzae	GCF_001053575^*a*^	AcrIIC4-Aca2	No self-target spacer
Pseudomonas citronellolis	GCF_001654435^*a*^	AcrIE4-IF7-Aca1	No self-target spacer
Pseudomonas aeruginosa strain C 1426	GCF_000412555^*a*^	AcrIF11-Aca1	No self-target spacer
Pseudomonas aeruginosa strain Jp54	GCF_003836565^*b*^	AcrIF6-Aca1	No self-target spacer
Vibrio parahaemolyticus	GCF_000736335^*a*^	AcrIF9-Aca1	No complete CRISPR-Cas
Shewanella xiamenensis	GCF_000712635^*a*^	AcrIF10-Aca1	No complete CRISPR-Cas
Neisseria meningitidis	GCF_001066195^*a*^	AcrIIC1-Aca3	No complete CRISPR-Cas
Neisseria meningitidis 992008	GCF_000724735^*a*^	AcrIIC2-AcaIIC3-Aca3	No complete CRISPR-Cas
Simonsiella muelleri ATCC 29453	GCF_000163775^*a*^	AcrIIC5-Aca2	No complete CRISPR-Cas
Pseudomonas aeruginosa strain S708_C14_RS	GCF_002136415^*b*^	AcrIE6-Aca1	No complete CRISPR-Cas
Pseudomonas aeruginosa strain 359	GCF_002312455^*b*^	AcrIF12-Aca4	No complete CRISPR-Cas

a These genomes are contained in the version of the RefSeq database that we have mined but have no self-targeting spacers according to the work of Watters et al. (12).

b These genomes were not in the version of the RefSeq database that we have mined, so we downloaded them separately from NCBI. All these genomes were analyzed using CRISPRCasFinder to confirm the presence of complete CRISPR-Cas systems and using our in-house programs to confirm the presence of self-targeting spacers.

This surprising finding is further confirmed by an analysis of the 1,193 Acr-Aca loci (Table 1). According to data from the work of Watters et al. (12), 376 (31.5%) out of the 1,193 Acr-Aca loci are from 370 (33.6% of 1,102) genomes with complete CRISPR-Cas systems and self-targeting CRISPR spacers (Fig. 2 and Table 1). Using CRISPRCasFinder, we further identified complete CRISPR-Cas loci in 212 additional bacterial genomes, and 113 (53.3%) of them have self-targeting spacers identified using our in-house programs. Therefore, 619 genomes (56.2%) of the 1,193 Acr-Aca loci do not have self-targeting spacers and 520 (47.2%) genomes do not have even CRISPR-Cas systems.

Furthermore, we have also analyzed the 975 unique Acr homologs (from 2,443 genomes) of RefSeq (regardless of having neighboring Aca homologs or not) and found that only 173 (17.7%) homologs are from genomes with self-targeting spacers according to data from Watters et al. (12). A CRISPRCasFinder search found that only 578 (23.7%) of the 2,443 genomes have complete CRISPR-Cas systems.

All these suggest that, although the presence of a self-targeting spacer is a strong indicator of the presence of Acr genes, searching for new Acrs should not be restricted to genomes with self-targeting spacers.

As mentioned above, 647 genomes (692 loci) of the 1,193 Acr-Aca loci are from P. aeruginosa. We found that 281 of these P. aeruginosa genomes (315 loci) do not have self-targeting spacers, and 211 genomes (232 loci) do not have even complete CRISPR-Cas systems. Similarly, out of the 250 L. monocytogenes genomes (297 loci) of the 1,193 Acr-Aca loci, 189 (208 loci) do not have self-targeting spacers, and 82 (85 loci) do not have complete CRISPR-Cas systems.

This finding of CRISPR-Cas systems present in some strains but absent in other strains of the same species is extremely interesting, as it revealed that CRISPR-Cas loci can be lost or gained rapidly among closely related genomes. As postulated in reference 14, the loss or erosion of CRISPR-Cas systems in bacterial genomes may be driven by the presence of Acrs located in MGEs, which cancel the selective pressure to maintain a functional CRISPR-Cas system.

Additionally, it is more surprising to find Acr-Aca loci in so many genomes without CRISPR-Cas systems, because Acrs should have no reason to exist if there are no CRISPR-Cas systems in the genome. However, this can be explained as the result of recent horizontal gene transfer of phages or other MGEs: the absence of CRISPR-Cas defense system in a bacterium makes it much easier for a phage to enter the cell and eventually become a prophage, which still carries the Acr-Aca loci that have been functional and essential in its previous hosts.

### Acr-Aca subtypes inferred by self-targeting spacers and Acr homology do not always agree.

According to the self-targeting idea, a genome with a complete CRISPR-Cas system and at least one CRISPR spacer targeting elsewhere in the self-genome will likely encode Acr proteins to avoid self-destruction. Therefore, one can infer a subtype for an Acr-Aca locus based on the Cas subtype of the self-targeting CRISPR spacer, with the assumption that the coexistence of the two elements in one genome indicates that the Acr-Aca locus inhibits the CRISPR-Cas system. The 376 Acr-Aca loci are from 370 genomes that have complete CRISPR-Cas systems and self-targeting spacers (Fig. 2 and Table 1), and the spacer targets are located on the same contig/chromosome as the Acr-Aca loci. Hence, two ways exist to infer subtypes for these 376 loci: (i) infer the subtype for each Acr-Aca locus based on what Cas subtype of self-targeting CRISPR-Cas system the genome has and (ii) infer the subtype based on Acr sequence homology, as each of the 376 Acr-Aca loci contains at least one Acr homolog, whose subtype can be assigned to the Acr-Aca locus.

When comparing the two Acr-Aca subtype assignment results, we found that 317 (84.3%) of the 376 loci received the same assignment from the two methods. Of the remaining 59 loci (Table 3), (a) 48 loci have the I-E assignment from self-targeting while containing homologs of AcrIF, (b) two loci have the I-F assignment from self-targeting while containing homologs of AcrIE, (c) eight loci have the I-B assignment from self-targeting while containing homologs of AcrIIA, and (d) one locus has the I-C assignment from self-targeting while containing homologs of AcrIE3 and IF4.

**TABLE 3:** Fifty-nine Acr-Aca loci with conflicting subtype assignments based on homology to known Acrs and on self-targeting spacers

No. of loci^*a*^	Homology to known Acrs	Self-targeting CRISPR-Cas subtype	No. of unique Acr homolog IDs
42	AcrIF3	I-E	1
4	AcrIF6	I-E	1
2	AcrIF1	I-E	1
2	AcrIE3	I-F	1
6	AcrIIA2	I-B	1
1	AcrIIA3, AcrIIA2	I-B	1
1	AcrIIA4	I-B	1
1	AcrIE3, AcrIF4	I-C	1

a Details about these loci can be found in Data Set S1.

However, the 376 Acr-Aca loci contain only 62 unique Acr homolog IDs (Table 1 and Fig. 2) and 361 (97.6%) out of the 370 genomes are from only two species: P. aeruginosa and L. monocytogenes. Therefore, many of these loci are identical duplicates containing the same protein IDs, as they are from closely related strain genomes. The 59 loci with conflicting subtype assignments actually correspond to only eight nonredundant loci with eight unique Acr homolog IDs (Table 3).

For these eight nonredundant loci, we believe that the homology-based assignments are more likely real, as the sequence identities of the eight homologs to known Acrs are all >85% (seven homologs are >95% identical). Loci of types a and b might not be surprising, as AcrIE and AcrIF homologs are often colocalized (Fig. 1). Loci of types c and d are from genomes with complete I-B or I-C CRISPR-Cas systems and self-targeting spacers, so these CRISPR-Cas systems must be turned off to avoid self-destruction, likely by other Acr-Aca loci of the genome (e.g., loci without homologs to known Acrs or loci located in different contigs/chromosomes than the one containing the self-targeting spacer targets). Indeed, genomes containing the 59 loci all have other loci passing filters 1 and 2, which nevertheless do not have homologs to known Acrs (first row in Table 1). On the other hand, loci of types c and d contain Acr homologs with high sequence identity to AcrIIA, AcrIE, and AcrIF proteins. As indicated above, genomes without CRISPR-Cas I-E, I-F, or II-A systems can still have Acr-Aca loci targeting these Cas subtypes due to recent gene transfer from other genomes or because the targeted CRISPR-Cas systems have degenerated in these genomes.

**FIG 4:**
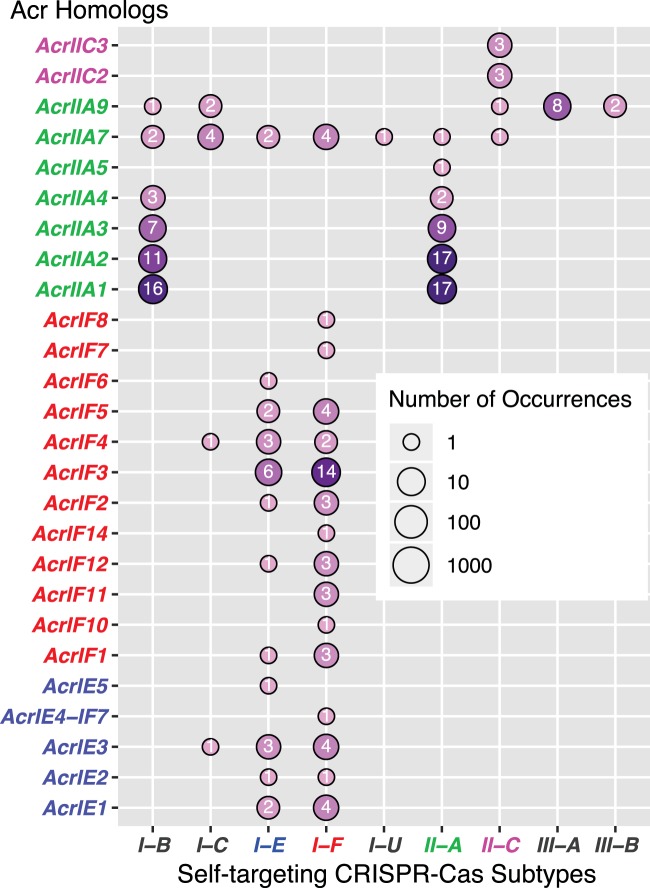
One hundred seventy-three Acr homologs are from genomes with self-targeting CRISPR-Cas spacers of different subtypes. The *y* axis shows 26 known Acr families, which have homologs in genomes with self-targeting CRISPR-Cas spacers. The Cas subtypes of the self-targeting spacers (according to the work of Watters et al. [12]) are shown on the *x* axis. The numbers in the circles are the numbers of Acr homologs.

In addition, as mentioned above, 173 out of the 973 Acr homologs (regardless of having neighboring Aca homologs or not) are found in genomes with self-targeting spacers. These 173 homologs contain the 62 homologs of the above 376 Acr-Aca loci. All the conflicting assignments observed for the 62 Acr homologs remain true for the 173 homologs (Fig. 4). Particularly, genomes with I-B CRISPR-Cas systems and self-targeting spacers contain various AcrIIA homologs. Additionally, although AcrIIA7 and AcrIIA9 have the most RefSeq homologs (Fig. 3A), only a small number of their homologs are found in genomes with self-targeting spacers (Fig. 4). More importantly, AcrIIA7 and AcrIIA9 homologs are found in genomes with various CRISPR-Cas subtypes and self-targeting spacers, but only one AcrIIA7 homolog is found in a genome with subtype II-A self-targeting spacers. All of these reveal that Acr homology-based and self-targeting spacer-based inferences for Acr subtypes do not always agree with each other, suggesting the complex evolutionary dynamics of the two systems (CRISPR-Cas and anti-CRISPR).

### New Acr subtypes are suggested by investigating genomic loci with self-targeting spacers.

Although the homology-based approach generated thousands of high-quality Acr homologs in RefSeq and IMG/VR databases, no new Acr families targeting new CRISPR-Cas subtypes can be (computationally) inferred. To this end, we have analyzed the 817 genomic loci (fourth column of data of Table 1) that are supported by the existence of a complete CRISPR-Cas system and self-targeting spacers in 672 genomes.

**FIG 5:**
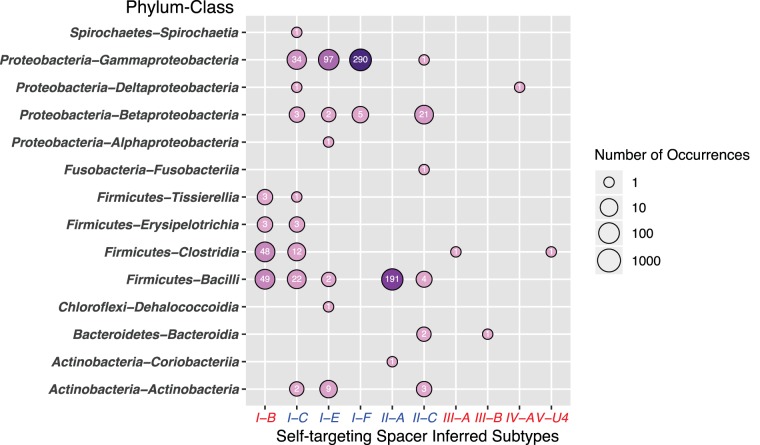
Eight hundred seventeen putative Acr-Aca loci are found in 14 bacterial classes and inferred to target 10 CRISPR-Cas subtypes. The bubble plot shows the phylum-class versus the CRISPR-Cas subtype of the 817 Acr-Aca loci supported by the existence of self-targeting spacers in the genomes. The circle size and color are coded in proportion to the number of loci in that circle (in logarithmic scale). The subtype of Acr-Aca loci is inferred from the CRISPR-Cas self-targeting spacers reported in the paper by Watters et al. (12).

The 817 Acr-Aca loci (Data Set S3) are from 113 species in total, with 429 (63.8%) genomes from the two species P. aeruginosa and L. monocytogenes. As a comparison, this percentage is 81.4% in the Acr homology-based 1,193 Acr-Aca loci and 97.6% for the 376 Acr-Aca loci having both Acr and Aca homologs as well as self-targeting spacers. Hence, the 817 Acr-Aca locus data set is less biased in terms of species distribution. Figure 5 shows that the 817 Acr-Aca loci are found in 14 bacterial classes of seven phyla: *Proteobacteria* (456 loci), *Firmicutes* (340 loci), *Actinobacteria* (15 loci), *Bacteroidetes* (three loci), *Chloroflexi* (one locus), *Fusobacteria* (one locus), and *Spirochaetes* (one locus).

10.1128/mSystems.00455-19.10 DATA SET S3 Eight hundred seventeen RefSeq genomic loci from genomes with self-targeting spacers. Download Data Set S3, XLS file, 1.1 MB. Copyright © 2019 Yin et al. 2019 Yin et al. This content is distributed under the terms of the Creative Commons Attribution 4.0 International license.

Inferred by self-targeting spacers in the genomes, the 817 Acr-Aca loci correspond to, in total, 10 subtypes of five types (Fig. 5), including five known subtypes (I-C, I-E, I-F, II-A, and II-C) for published Acr families and five newly discovered subtypes (I-B, III-A, III-B, IV-A, and V-U4). Of the new subtypes, I-B has 103 Acr-Aca loci from four *Firmicute* classes. This agrees with the fact that I-B is the most abundant CRISPR-Cas subtype in *Firmicutes* (34). However, each of the other four new subtypes contains only one locus (Table 4).

**TABLE 4:** Four putative Acr-Aca loci from four genomes with self-targeting spacers

Parameter	Data for species name^*a*^:			
	Pseudoramibacter alactolyticus ATCC 23263	Bacteroides salyersiae strain 2789STDY5608871	Geoalkalibacter subterraneus	Clostridioides difficile isolate VL_0239
RefSeq genome ID	GCF_000185505	GCF_001405695	GCF_000827125	GCF_900013625
Self-targeting CRISPR-Cas subtype	III-A	III-B	IV-A	V-U4
Acr-Aca locus on the same contig as spacer target	WP_006597765.1 to WP_006597766.1	WP_025819230.1 to WP_005928638.1	WP_040200104.1 to WP_040200103.1	WP_003438035.1 to WP_003438037.1
Aca/HTH protein ID	WP_006597766.1	WP_005928638.1	WP_040200103.1	WP_003438035.1
Aca is antitoxin hit	Yes	Yes	Yes	Yes
Acr protein ID	WP_006597765.1	WP_025819230.1	WP_040200104.1	WP_003438037.1
Acr is toxin hit	No	Yes	Yes	No
No. of other candidate Acr-Aca loci	0	0	0	4
Other CRISPR-Cas system(s)	I-C, II-A	No	I-C	I-B

a Details about these loci can be found in Data Set S3.

Specifically, Pseudoramibacter alactolyticus ATCC 23263 has a CRISPR-Cas system encoding a complete set of subtype III-A Cas enzymes according to data published in the work of Watters et al. (12). A CRISPRCasFinder search confirmed this but also found an I-C system and an II-A system (Table 4). Other than the Acr-Aca locus (WP_006597765.1 to WP_006597766.1) located on the same contig as the III-A spacer target, this genome does not have any other loci containing Aca or Acr homologs. Given that only the III-A system has a self-targeting spacer and that the genome has only one genomic locus that passed filters 1 and 2 (Table 2), it is likely that this single Acr-Aca locus inhibits the III-A system to prevent self-destruction rather than the I-C and II-A systems, which do not have self-targeting spacers in this genome.

Similarly, Clostridioides difficile isolate VL_0239 has a complete V-U4 CRISPR-Cas system and a self-targeting spacer target located on the same contig as the Acr-Aca locus (WP_003438035.1 to WP_003438037.1) according to the work of Watters et al. (12). This V-U4 system, however, was not found by the CRISPRCasFinder search, which instead identified an I-B system on a different contig (Table 4 and Data Set S3). Our Acr-Aca locus search also found four additional loci containing Aca homologs. Therefore, if the V-U4 system and its self-targeting spacer predicted by Watters et al. (12) are real, the V-U4 system is likely to be the target of the WP_003438035.1 to WP_003438037.1 locus. However, the system is also possibly inhibited by other Acr-Aca loci in the genome.

The third locus (WP_025819230.1 to WP_005928638.1) is from Bacteroides salyersiae strain 2789STDY5608871, which has a subtype III-B CRISPR-Cas system and a self-targeting spacer according to the work of Watters et al. (12). A CRISPRCasFinder search confirmed that it is the only complete CRISPR-Cas system in this genome. The Acr-Aca locus is also located close to the III-B CRISPR spacer target (15,500 bp apart on contig NZ_CYXS01000002.1 [Data Set S3]). However, a search against the TASmania database (35) found that the two proteins encoded by this locus were annotated as toxin (the Acr candidate WP_025819230.1, E value = 5.2e−10) and antitoxin (the Aca/HTH protein WP_005928638.1, E value = 0.001), respectively, which agrees with the RefSeq annotation of the two proteins: paired members of the type II toxin-antitoxin (TA) system RelE/ParE family.

The fourth locus (WP_040200104.1 to WP_040200103.1) is from Geoalkalibacter subterraneus, which has a complete subtype IV-A CRISPR-Cas system according to the work of Watters et al. (12). CRISPRCasFinder confirmed this and also identified a complete I-C system, which does not have a self-targeting spacer. The Acr-Aca locus was also annotated as a TA operon based on a search against the TASmania database.

We have further searched all the 817 Acr-Aca loci against the TASmania database and found that 32 (3.9%) of them have toxin and antitoxin hits in pairs. In all but one of the 32 loci, the antitoxin hit (HTH protein) was downstream of the toxin hit (Acr candidate). In addition to the two loci putatively targeting III-B and IV-A subtypes (Table 4), these 32 Acr-Aca loci also involve I-C (16 loci), I-F (eight loci), I-E (four loci), and II-C (two loci) subtypes.

As to the 103 I-B loci, they are from 57 genomes of 22 species. CRISPRCasFinder found that 45 (78.9%) of the 57 genomes do not have any other CRISPR-Cas systems than the I-B system (Data Set S3). The remaining 12 genomes (18 loci) also contain other CRISPR-Cas systems such as II-A (10 loci), I-C (nine loci), III-B (three loci), and III-A (one locus), which do not have self-targeting spacers in the genomes. Additionally, the TASmania database search did not find that any of these 103 loci have toxin and antitoxin pairs. Therefore, it is likely that most of these 103 loci inhibit the I-B CRISPR-Cas systems to avoid self-targeted DNA cleavage.

Of the five known subtypes, I-F and II-A subtypes have the largest numbers of loci (Fig. 5). Loci of these two subtypes also have the narrowest taxonomic distribution: most I-F loci are restricted to *Gammaproteobacteria* and all but one II-A locus are from *Bacilli*. The only non-*Bacilli* II-A locus is from *Eggerthella* sp. strain YY7918, which belongs to *Actinobacteria*. This species also has an I-C CRISPR-Cas system, which has no self-targeting spacer in the genome. The five non-*Gammaproteobacteria* I-F loci are from three *Delftia* species of *Betaproteobacteria* (Fig. 5 and Data Set S3), one of which also has an I-C CRISPR-Cas system, which has no self-targeting spacer in the genome.

The four published AcrIIC-Aca loci were from *Betaproteobacteria* and *Gammaproteobacteria* (Fig. 1). In addition to these two classes, Fig. 5 shows that there are 10 II-C loci from four new classes: *Actinobacteria* (three loci), *Bacteroidia* (two loci), *Bacilli* (four loci), and *Fusobacteriia* (one locus). Similarly, although published AcrIE-Aca loci were all from *Gammaproteobacteria* (Fig. 1), 15 I-E loci are found in five additional bacterial classes (Fig. 5).

Last, the I-C subtype had only one published protein, AcrIC1 (GenBank accession no. AKG19229.1), which is located in an operon with AcrVA genes in Moraxella bovoculi (Fig. 1). This AcrIC1 protein does not have any homologs in the RefSeq and IMG/VR databases that were searched in this study. Very interestingly, in our 817-locus data set, we found 79 I-C targeting loci distributed in nine different bacterial classes of four phyla (Fig. 5), making I-C the most widely distributed subtype. The finding of so many I-C-targeting Acr-Aca loci is in line with the previous finding that I-C is one of the most abundant CRISPR-Cas subtypes in bacteria, particularly overrepresented in *Firmicute*s and *Proteobacteria* (34).

CRISPRCasFinder search confirmed that most genomic loci of the five known subtypes are from genomes with no other CRISPR-Cas systems than the one with the self-targeting spacer. This suggests that these Acr-Aca loci should target their corresponding self-targeting CRISPR-Cas system, and thus the inferred subtypes in Fig. 5 are most likely real.

### Possible connection between anti-CRISPR systems and toxin-antitoxin systems.

It was interesting that toxin-antitoxin (TA) genes were found in the gene neighborhood of the Acr homologs (Table S4). It was more interesting that the TASmania database (35) search found that 3.9% of the 817 loci also matched the toxin and antitoxin in pairs. This percentage is 4.7% for the smallest, 508-locus data set and 4.3% for the largest, 53,216-locus data set (first column of data of Table 1). Although these matching loci might actually encode the TA system, it is most intriguing to recognize the compositional and functional similarity of the two systems: (i) both systems often require an HTH domain-containing protein (36), usually encoded by the first gene in the TA operon (as the antitoxin) whereas it is encoded by the second gene in the Acr-Aca operon (as the Aca); (ii) both systems tend to be found in prophages and other MGEs; (iii) the toxin protein produced by the TA system inhibits cell growth or induces programmed cell death (36), whereas the Acr protein turns off the cell’s CRISPR-Cas system, which could also lead to cell death. Therefore, one can speculate that these two systems might be evolutionarily related and functionally overlap to some extent, at least in some bacteria.

Furthermore, if the two systems are indeed related, the Aca proteins (equivalent of antitoxin), which are hypothesized to be regulator of Acrs, might play a role of negatively controlling the expression of Acr genes. Interestingly, a very recent paper experimentally proved that in an AcrIF8-Aca2 operon of the Pectobacterium carotovorum temperate phage ZF40, Aca2 served as a repressor of AcaIF8 (40). This might also explain why Acr genes found in lytic phages often do not have surrounding Aca genes, as they need to be immediately and highly expressed to turn off the host’s defense system to kill. This, however, may be happening in a more controlled manner with the negative control from Acas in lysogenic phages and prophages.

## MATERIALS AND METHODS

### Data sets.

The NCBI RefSeq database, which contained 75,599 bacterial genomes at that time, was downloaded on 27 August 2017. The IMG/VR database (28), which contained 760,453 assembled viral/proviral contigs at that time, was downloaded on 15 March 2019.

### Methods.

A bioinformatics pipeline (Fig. 2) was developed to process the RefSeq and IMG/VR genomic data using a list of filters to identify putative Acr-Aca loci. These filters essentially exploited sequence features extracted from a list of published Acr-Aca loci (http://bcb.unl.edu/AcrDB/Download/knownAcrAca/known-loci.xlsx; see also Text S1 in the supplemental material). This list contains representative proteins of 45 characterized Acr families as well as their associated Aca proteins.

In the data processing pipeline, two files of each RefSeq genome were processed: (i) a gene location file with the protein coding genes’ position and strand information in the DNA (i.e., the gff format file) and (ii) a protein sequence file (i.e., the faa format file). For the IMG/VR contigs, FragGeneScan (37) was run first on the nucleotide genome file (the fna format file) to generate the gene location file and protein sequence file.

The pipeline comprised the following steps.

(i) We used the published 45 Acr proteins (http://bcb.unl.edu/AcrDB/Download/knownAcrAca/Acrs/) as the query to search against 75,599 RefSeq bacterial genomes and 760,453 metagenome-assembled viral contigs (∼3% are from isolated phages or prophages) of the IMG/VR database. To qualify as Acr homologs, proteins have to meet the following criteria: (i) E value of <1e−2 to known Acr proteins, (ii) protein length of <200 amino acids (aa), and (iii) more importantly, Acr genes located in genomic loci (or operons) with all the genes encoding short proteins (<200 aa) on the same strand. Then, HTH domain-containing proteins (Acas) were searched for in the gene neighborhood of Acr homologs (Fig. 2 shows the criteria).

(ii) We then combined these new Aca proteins with the 39 previously published Aca proteins (Text S1), and in total 401 Aca proteins (http://bcb.unl.edu/AcrDB/Download/knownAcrAca/Acas/) were used as query to search against the 75,599 RefSeq bacterial genomes and the 760,453 metagenome-assembled viral contigs for Aca homologs (Fig. 2 shows the criteria). Then, we located the Aca homologs in genomic loci (or operons) encoding only short proteins (<200 aa) on the same strand, with short intergenic distances (<150 bp), and at least one gene encoding the Aca homolog. These are the strongest sequence features revealed in Text S1.

(iii) The genomic loci were then examined to see if they were located within or adjacent (±5kb) to mobile genetic elements (MGEs) such as prophages and genomic islands (GIs). Specifically, the genomic positions of the genomic loci were compared to the genomic locations of prophages in the PHASTER database (29) and to the genomic locations of GIs in the IslandViewer database (30).

(iv) The last step was to inspect if genomes with the genomic loci from the previous step also have complete CRISPR-Cas systems and self-targeting CRISPR arrays. Specifically, Watters et al. (12) identified 22,125 self-targeting cases in 9,155 bacterial genomes (available in Data S1 of the paper by Watters et al. [12]). Genomes from the previous step that were also included in this paper by Watters et al. (12) thus contain self-targeting spacers and their targets and were kept for further analysis. Some genomes have incomplete CRISPR-Cas loci, e.g., having only CRISPR arrays or having only Cas enzymes or missing some key Cas enzymes. Genomic loci from these genomes were removed. Additionally, only genomic loci that are colocalized with CRISPR self-targeted protospacers on the same contig/chromosomes were kept.
