# Supplementary material for: Bioinformatics Identification of Anti-CRISPR Loci by Using Homology, Guilt-by-Association, and CRISPR Self-Targeting Spacer Approaches
Source: mSystems. 2019 Sep 10;4(5):e00455-19. doi: 10.1128/mSystems.00455-19 (PMC6739104; doi:10.1128/mSystems.00455-19)
Supplement: TEXT S1 [file mSystems.00455-19-s0001.docx]

**Supplementary Results**

**Bioinformatics identification of anti-CRISPR loci using homology, guilt-by-association and CRISPR self-targeting approaches**

Yanbin Yin*^1,^*^#^, Bowen Yang*^1^*, and Sarah Entwistle*^2^*

*^1^ Nebraska Food for Health Center, Department of Food Science and Technology, University of Nebraska - Lincoln, Lincoln, NE 68588, USA*

*^2^ Department of Biological Sciences, Northern Illinois University, DeKalb, IL 60115, USA*

^#^ To whom correspondence should be addressed

Yanbin Yin

Tel: 1-402-472-4303

Email: [yyin@unl.edu](mailto:yyin@unl.edu)

**Running title:** Bioinformatics identification of anti-CRISPR loci

**Figure S1: Sequence features of published Acr-Aca loci**

**Figure S2: Phylogeny of 39 published Aca proteins**

**Table S7: Pfam domain search result of 36 published Aca proteins (total 39 Aca were searched but three did not have Pfam hits)**

**Table S8: The nine Aca families, their best Pfam HTH match, and co-localized Acr families**

**Sequence features of published Acrs**

The 45 Acr families include 14 AcrIF families (inhibiting I-F Cas proteins) ([1](#_ENREF_1), [2](#_ENREF_2)), seven AcrIE families ([2](#_ENREF_2), [3](#_ENREF_3)), one AcrIE/IF hybrid family ([2](#_ENREF_2)), one AcrIC family ([2](#_ENREF_2)), one AcrID family ([4](#_ENREF_4)), 11 AcrIIA families ([5-9](#_ENREF_5)), five AcrIIC families ([10](#_ENREF_10), [11](#_ENREF_11)), and five AcrVA families ([2](#_ENREF_2), [12](#_ENREF_12)) (http://bcb.unl.edu/AcrDB/Download/knownAcrAca/known-loci.xlsx and [https://tinyurl.com/anti-CRISPR](http://tinyurl.com/anti-CRISPR)). Among the 45 representative Acr proteins, 26 are from MGEs (mobile genomic elements such prophages and plasmids) of bacteria, five were discovered in environmental metagenomes, and the other 14 are from viruses. The 14 viruses include 11 lysogenic phages, two lytic phages, and one archaeal virus SIRV3.

Among the 40 Acr representative proteins (http://bcb.unl.edu/AcrDB/Download/knownAcrAca/known-loci.xlsx) characterized in isolated genomes, 30 have their encoding genes located immediately next or adjacent to an Aca gene in the genomes (**Figure S1-D and Figure 1**). The other 10 Acrs do not have neighboring Aca genes ([2](#_ENREF_2), [6](#_ENREF_6), [7](#_ENREF_7), [12](#_ENREF_12)): (i) two Acr proteins of the lytic phages (AcrIIA5 and AcrIIA6), and (ii) eight most recently characterized Acr proteins from *Moraxella* bacteria (AcrIF13, AcrIF14, AcrIC1, AcrVA1 through AcrVA5) (**Figure 1**). Notably, the AcrIIA1 is itself an Aca having an HTH domain. The five Acr genes very recently characterized in metagenomes (AcrIIA7 through AcrIIA11) do not have adjacent Aca genes either (**Figure 1**).

All these representative Acr and Aca genes are also on the same strand with short intergenic distances (**Figure S1-C and Figure 1**), and thus qualify for the concept of operons. Specifically, all the Acr-Aca intergenic distances are less than 150bp (**Figure S1-E**) except in the archaeal virus SIRV3 (Aca/YP_009272953.1 – AcrID1/YP_009272954.1 intergenic distance: 224bp) ([4](#_ENREF_4)). Interestingly, the SIRV3 Acr-Aca loci is also the only operon that has the Aca gene in the upstream of the Acr gene (AcrID), and all the other operons have the Aca gene in the downstream of Acr.

In addition, multiple tandem Acr genes can be present in one Acr-Aca operon (**Figure 1**), e.g., different AcrIE and AcrIF genes often coexist in one operon ([14](#_ENREF_14)). Furthermore, the newly found AcrVA genes, AcrIC1, AcrIF13, and AcrIF14 genes are also clustered in the *Moraxella* genomes ([2](#_ENREF_2)), although no HTH domain-containing Aca genes are around.

As previously reported, most Acr proteins are shorter than 150 aa (**Figure S1-A**). The seven exceptions include the five recently characterized Acr proteins from *Moraxella* bacteria (AcrIC1 [190 aa], AcrVA1 [170 aa], AcrVA2 [322 aa], AcrVA3 [168 aa], AcrVA4 [234 aa]) ([2](#_ENREF_2), [12](#_ENREF_12)), the AcrIIA6 (183 aa) from a lytic phage ([7](#_ENREF_7)), and the AcrIIA11 (182 aa) from human gut metagenome ([9](#_ENREF_9)). Notably, all these seven Acr proteins do not have nearby Aca genes either (**Figure 1**).

Lastly, 80% (36 out of 45) Acr proteins are acidic with isoelectric point lower than 7.0 (**Figure S1-F**). This is consistent with the finding that many Acr proteins bind to Cas proteins by mimicking DNAs ([4](#_ENREF_4), [15-18](#_ENREF_15)).

**Sequence features of published Acas**

We collected 39 Aca protein sequences from the literature, which include Aca proteins in http://bcb.unl.edu/AcrDB/Download/knownAcrAca/known-loci.xlsx plus Aca proteins identified in ([2](#_ENREF_2)). First of all, all published Aca proteins are shorter than 150 aa without exception (**Figure S1-B**). Secondly, all Aca proteins possess some variant of the HTH DNA-binding domain. The Pfam database contains 65 HTH domain models (with “HTH” in their Pfam short description) that are shorter than 150 aa (enlarging this threshold to 200 aa did not yield more families). We further added HTH_XRE from the SMART database and DUF1870 from the Pfam database to form an HTH hmmdb [hidden markov model (HMM) database]. This hmmdb was used to examine the published Aca proteins and their homologs for HTH domains.

Out of the 39 Aca proteins from literature, 36 of them matched in total 18 HTH domain models with an E-value < 0.01 (**Table S7**: DUF1870, HTH_11, HTH_17, HTH_19, HTH_23, HTH_24, HTH_26, HTH_28, HTH_29, HTH_3, HTH_31, HTH_36, HTH_37, HTH_40, HTH_7, HTH_8, HTH_Tnp_1, HTH_XRE). Note that one protein can have multiple overlapping HTH domain matches. When only keeping the best HTH match (i.e., with the lowest E-value) of each protein, nine HTH domains were found for the 36 proteins corresponding to nine families (**Table S8**). Each of the Aca family tends to form operons with different Acr genes, and correspond to different HTH domain models (**Table S8**) as the best match. There are also some HTH domains found to be the best match of different Aca families, e.g., HTH_XRE and HTH3.

In **Table S8**, among the nine families, Aca4 through Aca7 were very recently identified to be located next to AcrIF11 homologs in various bacteria ([2](#_ENREF_2)). In addition, the Aca protein (YP_009272953.1) identified in the archaeal virus SIRV3 was never officially named ([4](#_ENREF_4)). Here it is named Aca8 following the suggested Aca nomenclature ([https://tinyurl.com/anti-CRISPR](http://tinyurl.com/anti-CRISPR)) ([19](#_ENREF_19)). Lastly, AcrIIA1 and homologs also contain the HTH domain and tend to be clustered with other AcrIIA proteins, and thus qualify to be an Aca family as well.

Different Aca families share distant homology with the same evolutionary origin, as they all have the HTH structural fold and belong to the HTH clan/superfamily in the Pfam database. As expected, phylogenetic classification of the 39 Aca proteins supports the grouping of them into nine monophyletic families (**Figure S2**).

**Evaluation of the predicted Acr-Aca loci**

In this paper, we have focused on the study of three high-quality datasets resulted from our bioinformatics pipeline (**Figure 2**): (i) the 975 and 2,022 Acr homologs from RefSeq and IMG/VR respectively; (ii) the 1,193 RefSeq genomic loci containing both Acr homologs and Aca homologs; (iii) the 817 RefSeq genomic loci containing Aca homologs and from genomes with complete CRISPR-Cas systems and self-targeting spacers. However, the bioinformatics pipeline has actually generated a number of genomic loci datasets with different confidence levels according to what filters they have passed (**Table 1**). Apparently, more filtering steps mean less false positives but more false negatives. Indeed, if taking the 16 published bacterial Acr-Aca operons as the ground-truth dataset, the largest 53,216 loci dataset that passed the first two filters (2^nd^ column in **Table 1**) will have a recall=100% (no false negatives), while the smallest 508 loci dataset that passed all the filters (4^th^ column in **Table 1**) will have a recall=25% (see **Table 2**). However, given that most Acr-Aca loci in the RefSeq genomes have yet to be discovered and there is no true negative dataset available, it is not possible to calculate a precision for our pipeline.

In order to approximately evaluate the confidence level of these Acr-Aca datasets and discuss the usefulness of the three different approaches for new Acr-Aca loci identification, we have considered the following questions:

1. How many putative Acr-Aca loci does each genome have?

It is expected that one Acr-Aca locus is sufficient to turn off the CRISPR-Cas system of the genome. **Table 1** shows that only 4.2% (20/478) genomes of the smallest 508 loci dataset have >1 loci, meaning that most genomes contain only one Acr-Aca locus. As expected, this percentage gets higher in the other datasets (**Table 1**), as fewer filters were applied. For the largest 53,216 loci dataset, 14,405 (49.1%) genomes contain >1 loci. However, only 2,056 (7.0%) genomes have >3 loci, suggesting that most genomes have a very small number of loci. The genome with the largest number of loci is *Streptococcus pneumoniae* 09B02733 (GCF_001330115), which has 20 loci. However, most of these loci seem to be identical repeats in the genome.

However, we have noticed that even some published Acr-Aca loci are also from genomes containing > 1 loci. For example, in **Table 2**, the *Listeria monocytogenes* strain J0161 (GCF_000168635) contains two experimentally characterized loci: AcrIIA2-AcrIIA1 (NC_017545.1-2359954-2361564) and AcrIIA4-AcrIIA1 (NC_017545.1-2682416-2683133). In fact, this genome also contains two additional loci (NC_017545.1-460917-461532, NC_017545.1-795835-797632) that passed the filters 1 and 2. Another example is the *Pseudomonas aeruginosa* WH-SGI-V-07059 (GCF_001450485), which contains not only the characterized AcrIF4-AcrIE3-Aca1 locus but also two other loci that passed the filters 1 and 2. Therefore, we believe it might not be uncommon that one genome contain multiple Acr-Aca loci, and identifying new Acrs should not be limited to genomes with just one putative Acr-Aca locus.

2. How many genomes with the predicted Acr-Aca loci also have self-targeting spacers and complete CRISPR-Cas systems?

This question has been addressed in Results, which revealed probably the most surprising findings of this study. **Table 2** shows that only 25% of the published Acr-Aca loci are found in genomes with self-targeting spacers. This percentage is 31.5% for the 1,193 loci with both Acr and Aca homologs, and 17.7% for the 975 RefSeq Acr homologs (see Results). This means that the 508 loci and the 817 loci datasets (the 4^th^ and 5^th^ columns in **Table 1**) certainly have very high false negative rates, although the false positive rates might be lowest.

Additionally, 46.7% genomes of the published loci (**Table 2**) do not even have complete CRISPR-Cas systems, and this percentage is 47.2% and 76.3% for the 1,193 loci and the 975 Acr homologs, respectively. Hence, future experimental and computational identification of Acrs should also look at genomes without self-targeting spacers or even without complete CRISPR-Cas systems.

3. How many putative Acr-Aca loci contain homologs of known Acr proteins?

This question has also been addressed in Results. Sequence similarity to known Acr proteins is a strong indicator that the Acr-Aca loci identified by GBA or self-targeting spacers are likely to be real. **Table 1** shows that the more filters applied, the higher percentage of the predicted Acr-Aca loci with Acr homologs will be.

However, as revealed in **Table S2**, 28 (75.7%) of the known Acr families have homologs restricted to one single RefSeq bacterial class. In fact, only 15 (40.5%) of the known Acr families have homologs in more than one genus. Therefore, if we hope to find new Acr families, using homology-based approach will not help much, as the identified Acr homologs will be likely restricted to the same bacterial groups as the query Acr proteins (except for a small number of more conserved ones such as AcrIIA7 and AcrIIA9).

4. How similar are the Aca proteins to the published Aca proteins?

Finding reliable aca homologs are critical in the GBA approach to the new Acr-Aca loci identification. Therefore, we required that all Acr-Aca loci must have Aca homologs with >40% identity to the 401 Aca proteins compiled in step 1 (**Figure 1**). It is intuitive that loci with Aca homologs having higher similarity to these 401 Aca proteins will be more likely to be real. In this sense, the smallest 508 loci dataset will have the highest confidence level, as 73.4% (373) of these loci contain Aca proteins that are 100% identical to published Aca proteins. This percentage is 83.1% (422) for the 60% identity threshold. However, these high percentages are not surprising, because 367 (75.9%) of the 508 loci have both Acr and Aca homologs, among which 97.5% loci are from the two species *P. aeruginosa* and *L. monocytogenes*. Therefore, although the 508 loci dataset might have highest precision due to the presence of the large percentage of high similar Aca homologs, it is also the most biased in terms of species distribution and likely has the lowest recall.

1. Pawluk A, Staals RH, Taylor C, Watson BN, Saha S, Fineran PC, Maxwell KL, Davidson AR. 2016. Inactivation of CRISPR-Cas systems by anti-CRISPR proteins in diverse bacterial species. Nat Microbiol 1:16085.

2. Marino ND, Zhang JY, Borges AL, Sousa AA, Leon LM, Rauch BJ, Walton RT, Berry JD, Joung JK, Kleinstiver BP, Bondy-Denomy J. 2018. Discovery of widespread type I and type V CRISPR-Cas inhibitors. Science 362:240-242.

3. Pawluk A, Bondy-Denomy J, Cheung VH, Maxwell KL, Davidson AR. 2014. A new group of phage anti-CRISPR genes inhibits the type I-E CRISPR-Cas system of Pseudomonas aeruginosa. MBio 5:e00896.

4. He F, Bhoobalan-Chitty Y, Van LB, Kjeldsen AL, Dedola M, Makarova KS, Koonin EV, Brodersen DE, Peng X. 2018. Anti-CRISPR proteins encoded by archaeal lytic viruses inhibit subtype I-D immunity. Nat Microbiol 3:461-469.

5. Rauch BJ, Silvis MR, Hultquist JF, Waters CS, McGregor MJ, Krogan NJ, Bondy-Denomy J. 2017. Inhibition of CRISPR-Cas9 with Bacteriophage Proteins. Cell 168:150-158 e10.

6. Hynes AP, Rousseau GM, Lemay ML, Horvath P, Romero DA, Fremaux C, Moineau S. 2017. An anti-CRISPR from a virulent streptococcal phage inhibits Streptococcus pyogenes Cas9. Nat Microbiol 2:1374-1380.

7. Hynes AP, Rousseau GM, Agudelo D, Goulet A, Amigues B, Loehr J, Romero DA, Fremaux C, Horvath P, Doyon Y, Cambillau C, Moineau S. 2018. Widespread anti-CRISPR proteins in virulent bacteriophages inhibit a range of Cas9 proteins. Nat Commun 9:2919.

8. Uribe RV, van der Helm E, Misiakou MA, Lee SW, Kol S, Sommer MOA. 2019. Discovery and Characterization of Cas9 Inhibitors Disseminated across Seven Bacterial Phyla. Cell Host Microbe 25:233-241 e5.

9. Forsberg KJ, Bhatt IV, Schmidtke DT, Stoddard BL, Kaiser BK, Malik HS. 2019. Functional metagenomics-guided discovery of potent Cas9 inhibitors in the human microbiome. bioRxiv doi:10.1101/569095:569095.

10. Pawluk A, Amrani N, Zhang Y, Garcia B, Hidalgo-Reyes Y, Lee J, Edraki A, Shah M, Sontheimer EJ, Maxwell KL, Davidson AR. 2016. Naturally Occurring Off-Switches for CRISPR-Cas9. Cell 167:1829-1838 e9.

11. Lee J, Mir A, Edraki A, Garcia B, Amrani N, Lou HE, Gainetdinov I, Pawluk A, Ibraheim R, Gao XD, Liu P, Davidson AR, Maxwell KL, Sontheimer EJ. 2018. Potent Cas9 Inhibition in Bacterial and Human Cells by AcrIIC4 and AcrIIC5 Anti-CRISPR Proteins. MBio 9.

12. Watters KE, Fellmann C, Bai HB, Ren SM, Doudna JA. 2018. Systematic discovery of natural CRISPR-Cas12a inhibitors. Science 362:236-239.

13. Harrison KJ, Crecy-Lagard V, Zallot R. 2018. Gene Graphics: a genomic neighborhood data visualization web application. Bioinformatics 34:1406-1408.

14. Bondy-Denomy J. 2018. Protein Inhibitors of CRISPR-Cas9. ACS Chem Biol 13:417-423.

15. Bondy-Denomy J, Garcia B, Strum S, Du M, Rollins MF, Hidalgo-Reyes Y, Wiedenheft B, Maxwell KL, Davidson AR. 2015. Multiple mechanisms for CRISPR-Cas inhibition by anti-CRISPR proteins. Nature 526:136-9.

16. Shin J, Jiang F, Liu JJ, Bray NL, Rauch BJ, Baik SH, Nogales E, Bondy-Denomy J, Corn JE, Doudna JA. 2017. Disabling Cas9 by an anti-CRISPR DNA mimic. Sci Adv 3:e1701620.

17. Dong, Guo M, Wang S, Zhu Y, Wang S, Xiong Z, Yang J, Xu Z, Huang Z. 2017. Structural basis of CRISPR-SpyCas9 inhibition by an anti-CRISPR protein. Nature 546:436-439.

18. Liu L, Yin M, Wang M, Wang Y. 2018. Phage AcrIIA2 DNA Mimicry: Structural Basis of the CRISPR and Anti-CRISPR Arms Race. Mol Cell doi:10.1016/j.molcel.2018.11.011.

19. Bondy-Denomy J, Davidson AR, Doudna JA, Fineran PC, Maxwell KL, Moineau S, Peng X, Sontheimer EJ, Wiedenheft B. 2018. A Unified Resource for Tracking Anti-CRISPR Names. The CRISPR Journal 1:304-305.

20. Katoh K, Standley DM. 2013. MAFFT multiple sequence alignment software version 7: improvements in performance and usability. Mol Biol Evol 30:772-80.

21. Price MN, Dehal PS, Arkin AP. 2010. FastTree 2--approximately maximum-likelihood trees for large alignments. PLoS One 5:e9490.

22. Letunic I, Bork P. 2016. Interactive tree of life (iTOL) v3: an online tool for the display and annotation of phylogenetic and other trees. Nucleic Acids Res 44:W242-5.

**Figure S1: Sequence features of published Acr-Aca loci**

The data used to make all the plots can be found in http://bcb.unl.edu/AcrDB/Download/knownAcrAca/known-loci.xlsx. For (B), the 39 Aca protein GenBank IDs can be found in **Figure S2**, which also includes Aca4 through Aca7 proteins identified in ([2](#_ENREF_2)). For (E), the total number is 30, because there are 10 Acr genes without neighboring Aca genes [see (D)]. (A) and (F) include the five Acr proteins discovered in metagenomes.


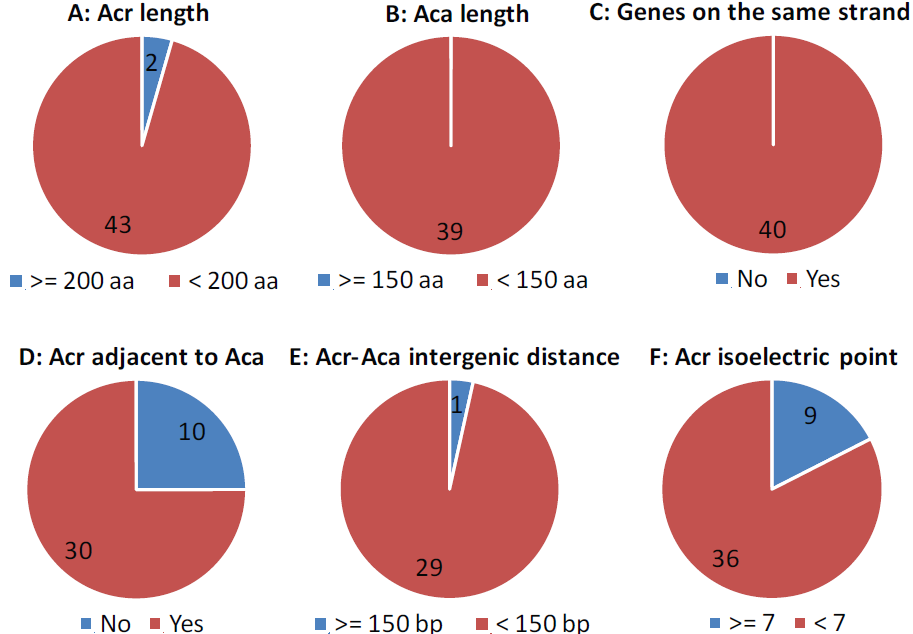


**Figure S2: Phylogeny of 39 published Aca proteins**

The 39 protein sequences were aligned with MAFFT with the mafft-linsi method ([20](#_ENREF_20)). The aligned sequences were used to build a approximately-maximum-likelihood phylogeny using FastTree ([21](#_ENREF_21)). The phylogeny was then visualized in the iTOL (Interactive Tree Of Life) web server ([22](#_ENREF_22)). In the phylogram, for AcrIF11 linked Aca families (e.g., Aca4 through Aca7), the protein labels are in “AcrIF11|Aca #|Aca protein ID” format, because they were identified by homology searching of AcrIF11 homologs in different bacteria ([2](#_ENREF_2)). For the other Aca families, the protein labels are in “Acr # - Aca #|Aca protein ID” format, except for Aca8-AcrID1 reflecting the fact that Aca8 is in the upstream of AcrID1. Supporting values > 0.8 are shown beside the nodes.


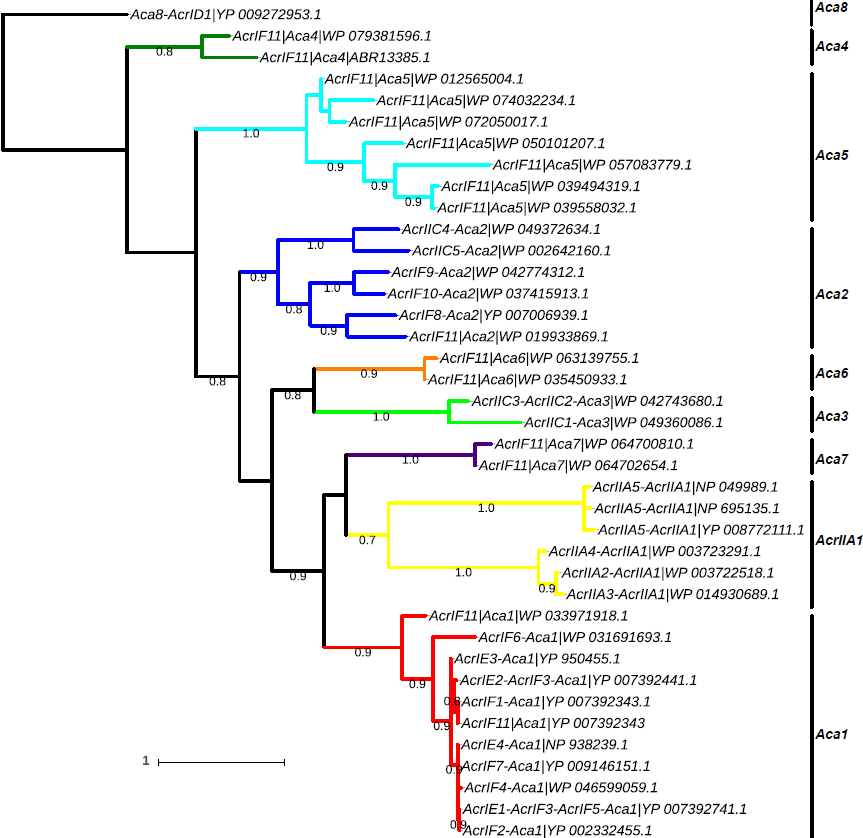


**Table S7: Pfam domain search result of 36 published Aca proteins (total 39 Aca were searched but three did not have Pfam hits)**

| Aca Protein | Length | Start | End | E-value | HTH domain | Length | Start | End |
| --- | --- | --- | --- | --- | --- | --- | --- | --- |
| Aca1\|WP_033971918.1 | 67 | 15 | 47 | 3.20E-08 | HTH_XRE | 56 | 2 | 34 |
| Aca1\|WP_033971918.1 | 67 | 13 | 49 | 1.30E-07 | HTH_31 | 64 | 4 | 37 |
| Aca1\|WP_033971918.1 | 67 | 20 | 45 | 1.50E-06 | HTH_3 | 55 | 6 | 31 |
| Aca1\|WP_033971918.1 | 67 | 19 | 46 | 4.00E-05 | HTH_23 | 50 | 14 | 40 |
| Aca1\|WP_033971918.1 | 67 | 19 | 46 | 8.10E-05 | HTH_19 | 65 | 8 | 35 |
| Aca1\|WP_033971918.1 | 67 | 20 | 41 | 0.00016 | HTH_8 | 42 | 15 | 36 |
| Aca1\|WP_033971918.1 | 67 | 22 | 56 | 0.00016 | HTH_Tnp_1 | 75 | 22 | 56 |
| Aca1\|WP_033971918.1 | 67 | 18 | 45 | 0.00069 | HTH_24 | 48 | 12 | 39 |
| Aca1\|YP_007392343 | 79 | 24 | 48 | 2.30E-05 | HTH_8 | 42 | 13 | 37 |
| Aca1\|YP_007392343 | 79 | 24 | 58 | 3.10E-05 | HTH_24 | 48 | 12 | 46 |
| Aca1\|YP_007392343 | 79 | 22 | 43 | 0.00016 | HTH_3 | 55 | 2 | 23 |
| Aca1\|YP_007392343 | 79 | 20 | 49 | 0.00021 | HTH_31 | 64 | 5 | 34 |
| Aca1\|YP_007392343 | 79 | 20 | 51 | 0.00025 | HTH_19 | 65 | 3 | 30 |
| Aca1\|YP_007392343 | 79 | 21 | 44 | 0.00038 | HTH_XRE | 56 | 2 | 25 |
| Aca2\|WP_019933869.1 | 125 | 1 | 113 | 8.20E-31 | DUF1870 | 118 | 1 | 114 |
| Aca2\|WP_019933869.1 | 125 | 5 | 47 | 2.30E-06 | HTH_31 | 64 | 5 | 47 |
| Aca2\|WP_019933869.1 | 125 | 5 | 48 | 5.90E-05 | HTH_XRE | 56 | 1 | 43 |
| Aca2\|WP_019933869.1 | 125 | 6 | 46 | 0.0002 | HTH_3 | 55 | 1 | 40 |
| Aca3\|WP_049360086.1 | 70 | 15 | 48 | 4.80E-08 | HTH_19 | 65 | 3 | 35 |
| Aca3\|WP_049360086.1 | 70 | 16 | 47 | 7.30E-08 | HTH_XRE | 56 | 2 | 32 |
| Aca3\|WP_049360086.1 | 70 | 16 | 46 | 2.20E-07 | HTH_3 | 55 | 1 | 30 |
| Aca3\|WP_049360086.1 | 70 | 16 | 49 | 3.90E-06 | HTH_31 | 64 | 6 | 38 |
| Aca4\|ABR13385.1 | 67 | 23 | 60 | 2.40E-05 | HTH_23 | 50 | 10 | 47 |
| Aca4\|ABR13385.1 | 67 | 9 | 50 | 0.00011 | HTH_XRE | 56 | 2 | 29 |
| Aca4\|ABR13385.1 | 67 | 31 | 50 | 0.00052 | HTH_3 | 55 | 9 | 28 |
| Aca4\|ABR13385.1 | 67 | 32 | 53 | 0.00056 | HTH_24 | 48 | 18 | 39 |
| Aca4\|ABR13385.1 | 67 | 31 | 57 | 0.00086 | HTH_29 | 63 | 11 | 38 |
| Aca8-AcrID1\|YP_009272953.1 | 55 | 27 | 53 | 2.30E-05 | HTH_23 | 50 | 13 | 39 |
| Aca8-AcrID1\|YP_009272953.1 | 55 | 27 | 54 | 2.70E-05 | HTH_29 | 63 | 7 | 35 |
| Aca8-AcrID1\|YP_009272953.1 | 55 | 26 | 53 | 8.30E-05 | HTH_7 | 45 | 16 | 43 |
| Aca8-AcrID1\|YP_009272953.1 | 55 | 23 | 55 | 0.00024 | HTH_40 | 89 | 4 | 36 |
| Aca8-AcrID1\|YP_009272953.1 | 55 | 27 | 52 | 0.00024 | HTH_XRE | 56 | 6 | 31 |
| Aca8-AcrID1\|YP_009272953.1 | 55 | 28 | 53 | 0.00046 | HTH_11 | 55 | 12 | 37 |
| Aca8-AcrID1\|YP_009272953.1 | 55 | 33 | 52 | 0.00065 | HTH_17 | 51 | 3 | 22 |
| Aca4\|WP_079381596.1 | 72 | 22 | 60 | 3.60E-05 | HTH_23 | 50 | 9 | 47 |
| Aca4\|WP_079381596.1 | 72 | 29 | 58 | 5.80E-05 | HTH_29 | 63 | 9 | 39 |
| Aca4\|WP_079381596.1 | 72 | 22 | 53 | 0.00034 | HTH_28 | 52 | 4 | 34 |
| Aca5\|WP_012565004.1 | 68 | 18 | 40 | 4.80E-07 | HTH_28 | 52 | 12 | 34 |
| Aca5\|WP_012565004.1 | 68 | 20 | 41 | 3.90E-05 | HTH_3 | 55 | 11 | 32 |
| Aca5\|WP_012565004.1 | 68 | 21 | 43 | 5.30E-05 | HTH_XRE | 56 | 13 | 35 |
| Aca5\|WP_012565004.1 | 68 | 17 | 37 | 7.20E-05 | HTH_8 | 42 | 17 | 37 |
| Aca5\|WP_012565004.1 | 68 | 20 | 40 | 7.70E-05 | HTH_23 | 50 | 19 | 39 |
| Aca5\|WP_012565004.1 | 68 | 20 | 40 | 0.00022 | HTH_36 | 55 | 27 | 47 |
| Aca5\|WP_039558032.1 | 60 | 12 | 34 | 0.00042 | HTH_28 | 52 | 12 | 34 |
| Aca5\|WP_050101207.1 | 64 | 16 | 34 | 3.70E-05 | HTH_28 | 52 | 16 | 34 |
| Aca5\|WP_050101207.1 | 64 | 15 | 34 | 0.00082 | HTH_36 | 55 | 28 | 47 |
| Aca5\|WP_072050017.1 | 65 | 16 | 37 | 5.60E-06 | HTH_28 | 52 | 13 | 34 |
| Aca5\|WP_072050017.1 | 65 | 17 | 38 | 3.40E-05 | HTH_3 | 55 | 11 | 32 |
| Aca5\|WP_072050017.1 | 65 | 14 | 33 | 0.00011 | HTH_8 | 42 | 17 | 36 |
| Aca5\|WP_072050017.1 | 65 | 17 | 37 | 0.00021 | HTH_36 | 55 | 27 | 47 |
| Aca5\|WP_072050017.1 | 65 | 17 | 37 | 0.00023 | HTH_23 | 50 | 19 | 39 |
| Aca5\|WP_072050017.1 | 65 | 18 | 38 | 0.00026 | HTH_XRE | 56 | 13 | 33 |
| Aca5\|WP_072050017.1 | 65 | 17 | 38 | 0.00049 | HTH_26 | 63 | 12 | 33 |
| Aca5\|WP_074032234.1 | 72 | 21 | 45 | 1.70E-05 | HTH_3 | 55 | 9 | 33 |
| Aca5\|WP_074032234.1 | 72 | 23 | 47 | 2.50E-05 | HTH_XRE | 56 | 12 | 36 |
| Aca5\|WP_074032234.1 | 72 | 21 | 43 | 5.40E-05 | HTH_28 | 52 | 12 | 34 |
| Aca6\|WP_035450933.1 | 65 | 4 | 37 | 4.00E-10 | HTH_3 | 55 | 1 | 34 |
| Aca6\|WP_035450933.1 | 65 | 4 | 54 | 9.40E-10 | HTH_XRE | 56 | 2 | 47 |
| Aca6\|WP_035450933.1 | 65 | 5 | 38 | 2.80E-09 | HTH_31 | 64 | 7 | 41 |
| Aca6\|WP_035450933.1 | 65 | 7 | 35 | 2.50E-08 | HTH_23 | 50 | 14 | 40 |
| Aca6\|WP_035450933.1 | 65 | 3 | 34 | 5.60E-05 | HTH_28 | 52 | 5 | 34 |
| Aca6\|WP_035450933.1 | 65 | 10 | 28 | 0.00026 | HTH_8 | 42 | 16 | 34 |
| Aca6\|WP_035450933.1 | 65 | 5 | 35 | 0.0004 | HTH_19 | 65 | 5 | 35 |
| Aca6\|WP_035450933.1 | 65 | 2 | 34 | 0.00049 | HTH_37 | 80 | 21 | 53 |
| Aca6\|WP_063139755.1 | 64 | 5 | 37 | 4.30E-10 | HTH_3 | 55 | 2 | 34 |
| Aca6\|WP_063139755.1 | 64 | 5 | 39 | 2.60E-09 | HTH_31 | 64 | 7 | 42 |
| Aca6\|WP_063139755.1 | 64 | 5 | 53 | 3.60E-09 | HTH_XRE | 56 | 3 | 46 |
| Aca6\|WP_063139755.1 | 64 | 8 | 34 | 7.70E-08 | HTH_23 | 50 | 15 | 39 |
| Aca6\|WP_063139755.1 | 64 | 11 | 34 | 0.0002 | HTH_28 | 52 | 11 | 34 |
| Aca6\|WP_063139755.1 | 64 | 11 | 28 | 0.00032 | HTH_8 | 42 | 17 | 34 |
| Aca6\|WP_063139755.1 | 64 | 3 | 35 | 0.00046 | HTH_37 | 80 | 22 | 54 |
| Aca6\|WP_063139755.1 | 64 | 8 | 37 | 0.00067 | HTH_19 | 65 | 8 | 37 |
| Aca7\|WP_064700810.1 | 68 | 26 | 42 | 0.00012 | HTH_3 | 55 | 11 | 27 |
| Aca7\|WP_064700810.1 | 68 | 26 | 42 | 0.00067 | HTH_XRE | 56 | 12 | 28 |
| Aca7\|WP_064702654.1 | 68 | 26 | 42 | 9.20E-06 | HTH_3 | 55 | 11 | 27 |
| Aca7\|WP_064702654.1 | 68 | 26 | 44 | 2.90E-05 | HTH_XRE | 56 | 12 | 30 |
| Aca7\|WP_064702654.1 | 68 | 26 | 55 | 7.30E-05 | HTH_31 | 64 | 16 | 46 |
| Aca7\|WP_064702654.1 | 68 | 26 | 51 | 9.80E-05 | HTH_19 | 65 | 14 | 39 |
| Aca7\|WP_064702654.1 | 68 | 15 | 41 | 0.00012 | HTH_8 | 42 | 8 | 35 |
| Aca7\|WP_064702654.1 | 68 | 26 | 39 | 0.00025 | HTH_11 | 55 | 17 | 30 |
| Aca7\|WP_064702654.1 | 68 | 26 | 39 | 0.00096 | HTH_24 | 48 | 19 | 32 |
| AcrIE1-AcrIF3-AcrIF5-Aca1\|YP_007392741.1 | 73 | 18 | 42 | 2.00E-05 | HTH_8 | 42 | 13 | 37 |
| AcrIE1-AcrIF3-AcrIF5-Aca1\|YP_007392741.1 | 73 | 18 | 52 | 2.90E-05 | HTH_24 | 48 | 12 | 46 |
| AcrIE1-AcrIF3-AcrIF5-Aca1\|YP_007392741.1 | 73 | 16 | 37 | 0.00013 | HTH_3 | 55 | 2 | 23 |
| AcrIE1-AcrIF3-AcrIF5-Aca1\|YP_007392741.1 | 73 | 14 | 42 | 0.00017 | HTH_31 | 64 | 5 | 33 |
| AcrIE1-AcrIF3-AcrIF5-Aca1\|YP_007392741.1 | 73 | 15 | 39 | 0.00029 | HTH_XRE | 56 | 2 | 26 |
| AcrIE1-AcrIF3-AcrIF5-Aca1\|YP_007392741.1 | 73 | 14 | 45 | 0.0003 | HTH_19 | 65 | 3 | 30 |
| AcrIE2-AcrIF3-Aca1\|YP_007392441.1 | 74 | 19 | 43 | 6.00E-05 | HTH_8 | 42 | 13 | 37 |
| AcrIE2-AcrIF3-Aca1\|YP_007392441.1 | 74 | 17 | 37 | 0.00062 | HTH_3 | 55 | 2 | 22 |
| AcrIE3-Aca1\|YP_950455.1 | 73 | 18 | 42 | 2.00E-05 | HTH_8 | 42 | 13 | 37 |
| AcrIE3-Aca1\|YP_950455.1 | 73 | 18 | 52 | 2.50E-05 | HTH_24 | 48 | 12 | 46 |
| AcrIE3-Aca1\|YP_950455.1 | 73 | 16 | 37 | 0.00013 | HTH_3 | 55 | 2 | 23 |
| AcrIE3-Aca1\|YP_950455.1 | 73 | 14 | 43 | 0.00017 | HTH_31 | 64 | 5 | 34 |
| AcrIE3-Aca1\|YP_950455.1 | 73 | 14 | 45 | 0.0002 | HTH_19 | 65 | 3 | 30 |
| AcrIE3-Aca1\|YP_950455.1 | 73 | 15 | 39 | 0.0003 | HTH_XRE | 56 | 2 | 26 |
| AcrIF10-Aca2\|WP_037415913.1 | 120 | 1 | 108 | 3.50E-21 | DUF1870 | 118 | 1 | 112 |
| AcrIF10-Aca2\|WP_037415913.1 | 120 | 5 | 57 | 2.40E-06 | HTH_31 | 64 | 5 | 58 |
| AcrIF1-Aca1\|YP_007392343.1 | 79 | 24 | 48 | 2.30E-05 | HTH_8 | 42 | 13 | 37 |
| AcrIF1-Aca1\|YP_007392343.1 | 79 | 24 | 58 | 3.10E-05 | HTH_24 | 48 | 12 | 46 |
| AcrIF1-Aca1\|YP_007392343.1 | 79 | 22 | 43 | 0.00016 | HTH_3 | 55 | 2 | 23 |
| AcrIF1-Aca1\|YP_007392343.1 | 79 | 20 | 49 | 0.00021 | HTH_31 | 64 | 5 | 34 |
| AcrIF1-Aca1\|YP_007392343.1 | 79 | 20 | 51 | 0.00025 | HTH_19 | 65 | 3 | 30 |
| AcrIF1-Aca1\|YP_007392343.1 | 79 | 21 | 44 | 0.00038 | HTH_XRE | 56 | 2 | 25 |
| AcrIF4-Aca1\|WP_046599059.1 | 82 | 27 | 51 | 2.50E-05 | HTH_8 | 42 | 13 | 37 |
| AcrIF4-Aca1\|WP_046599059.1 | 82 | 27 | 61 | 3.90E-05 | HTH_24 | 48 | 12 | 46 |
| AcrIF4-Aca1\|WP_046599059.1 | 82 | 25 | 46 | 0.00018 | HTH_3 | 55 | 2 | 23 |
| AcrIF4-Aca1\|WP_046599059.1 | 82 | 23 | 51 | 0.00024 | HTH_31 | 64 | 5 | 33 |
| AcrIF4-Aca1\|WP_046599059.1 | 82 | 24 | 48 | 0.00039 | HTH_XRE | 56 | 2 | 26 |
| AcrIF4-Aca1\|WP_046599059.1 | 82 | 23 | 54 | 0.0004 | HTH_19 | 65 | 3 | 30 |
| AcrIF6-Aca1\|WP_031691693.1 | 72 | 13 | 46 | 1.50E-05 | HTH_31 | 64 | 4 | 37 |
| AcrIF6-Aca1\|WP_031691693.1 | 72 | 22 | 45 | 1.60E-05 | HTH_29 | 63 | 10 | 34 |
| AcrIF6-Aca1\|WP_031691693.1 | 72 | 21 | 45 | 5.50E-05 | HTH_24 | 48 | 15 | 39 |
| AcrIF6-Aca1\|WP_031691693.1 | 72 | 15 | 46 | 0.00024 | HTH_37 | 80 | 23 | 54 |
| AcrIF6-Aca1\|WP_031691693.1 | 72 | 19 | 38 | 0.00056 | HTH_23 | 50 | 14 | 32 |
| AcrIF6-Aca1\|WP_031691693.1 | 72 | 20 | 41 | 0.00059 | HTH_8 | 42 | 15 | 36 |
| AcrIF7-Aca1\|YP_009146151.1 | 73 | 18 | 42 | 2.00E-05 | HTH_8 | 42 | 13 | 37 |
| AcrIF7-Aca1\|YP_009146151.1 | 73 | 18 | 52 | 2.90E-05 | HTH_24 | 48 | 12 | 46 |
| AcrIF7-Aca1\|YP_009146151.1 | 73 | 16 | 37 | 0.00013 | HTH_3 | 55 | 2 | 23 |
| AcrIF7-Aca1\|YP_009146151.1 | 73 | 14 | 42 | 0.00017 | HTH_31 | 64 | 5 | 33 |
| AcrIF7-Aca1\|YP_009146151.1 | 73 | 14 | 45 | 0.00027 | HTH_19 | 65 | 3 | 30 |
| AcrIF7-Aca1\|YP_009146151.1 | 73 | 15 | 39 | 0.00029 | HTH_XRE | 56 | 2 | 26 |
| AcrIF8-Aca2\|YP_007006939.1 | 116 | 1 | 112 | 4.00E-31 | DUF1870 | 118 | 1 | 114 |
| AcrIF8-Aca2\|YP_007006939.1 | 116 | 5 | 43 | 2.40E-05 | HTH_31 | 64 | 5 | 42 |
| AcrIF8-Aca2\|YP_007006939.1 | 116 | 5 | 46 | 0.00016 | HTH_XRE | 56 | 1 | 37 |
| AcrIF9-Aca2\|WP_042774312.1 | 119 | 1 | 106 | 5.70E-24 | DUF1870 | 118 | 1 | 112 |
| AcrIF9-Aca2\|WP_042774312.1 | 119 | 4 | 53 | 4.30E-05 | HTH_31 | 64 | 4 | 54 |
| AcrIIA2-AcrIIA1\|WP_003722518.1 | 149 | 6 | 60 | 4.00E-06 | HTH_26 | 63 | 2 | 56 |
| AcrIIA2-AcrIIA1\|WP_003722518.1 | 149 | 6 | 32 | 3.50E-05 | HTH_XRE | 56 | 2 | 28 |
| AcrIIA2-AcrIIA1\|WP_003722518.1 | 149 | 8 | 31 | 5.80E-05 | HTH_3 | 55 | 3 | 26 |
| AcrIIA3-AcrIIA1\|WP_014930689.1 | 149 | 6 | 55 | 4.60E-06 | HTH_26 | 63 | 2 | 51 |
| AcrIIA3-AcrIIA1\|WP_014930689.1 | 149 | 6 | 32 | 3.50E-05 | HTH_XRE | 56 | 2 | 28 |
| AcrIIA3-AcrIIA1\|WP_014930689.1 | 149 | 8 | 31 | 5.80E-05 | HTH_3 | 55 | 3 | 26 |
| AcrIIA4-AcrIIA1\|WP_003723291.1 | 149 | 6 | 49 | 2.20E-07 | HTH_26 | 63 | 2 | 45 |
| AcrIIA4-AcrIIA1\|WP_003723291.1 | 149 | 6 | 51 | 3.70E-07 | HTH_XRE | 56 | 2 | 46 |
| AcrIIA4-AcrIIA1\|WP_003723291.1 | 149 | 8 | 34 | 8.00E-06 | HTH_3 | 55 | 3 | 29 |
| AcrIIA5-AcrIIA1\|NP_049989.1 | 140 | 20 | 53 | 1.00E-05 | HTH_3 | 55 | 13 | 46 |
| AcrIIA5-AcrIIA1\|NP_049989.1 | 140 | 19 | 53 | 1.70E-05 | HTH_26 | 63 | 13 | 45 |
| AcrIIA5-AcrIIA1\|NP_049989.1 | 140 | 22 | 54 | 6.80E-05 | HTH_XRE | 56 | 16 | 45 |
| AcrIIA5-AcrIIA1\|NP_695135.1 | 140 | 14 | 53 | 0.00017 | HTH_26 | 63 | 8 | 45 |
| AcrIIA5-AcrIIA1\|NP_695135.1 | 140 | 20 | 53 | 0.00024 | HTH_3 | 55 | 13 | 46 |
| AcrIIA5-AcrIIA1\|NP_695135.1 | 140 | 22 | 54 | 0.001 | HTH_XRE | 56 | 16 | 45 |
| AcrIIA5-AcrIIA1\|YP_008772111.1 | 140 | 14 | 54 | 2.50E-06 | HTH_26 | 63 | 8 | 46 |
| AcrIIA5-AcrIIA1\|YP_008772111.1 | 140 | 20 | 49 | 2.30E-05 | HTH_3 | 55 | 13 | 42 |
| AcrIIA5-AcrIIA1\|YP_008772111.1 | 140 | 21 | 51 | 0.00011 | HTH_XRE | 56 | 15 | 42 |
| AcrIIA5-AcrIIA1\|YP_008772111.1 | 140 | 11 | 35 | 0.0016 | HTH_11 | 55 | 9 | 34 |
| AcrIIC1-Aca3\|WP_049360086.1 | 70 | 15 | 48 | 4.80E-08 | HTH_19 | 65 | 3 | 35 |
| AcrIIC1-Aca3\|WP_049360086.1 | 70 | 16 | 47 | 7.30E-08 | HTH_XRE | 56 | 2 | 32 |
| AcrIIC1-Aca3\|WP_049360086.1 | 70 | 16 | 46 | 2.20E-07 | HTH_3 | 55 | 1 | 30 |
| AcrIIC1-Aca3\|WP_049360086.1 | 70 | 16 | 49 | 3.90E-06 | HTH_31 | 64 | 6 | 38 |
| AcrIIC3-AcrIIC2-Aca3\|WP_042743680.1 | 69 | 14 | 44 | 8.20E-09 | HTH_XRE | 56 | 2 | 32 |
| AcrIIC3-AcrIIC2-Aca3\|WP_042743680.1 | 69 | 14 | 45 | 1.50E-08 | HTH_3 | 55 | 1 | 32 |
| AcrIIC3-AcrIIC2-Aca3\|WP_042743680.1 | 69 | 13 | 45 | 1.60E-06 | HTH_19 | 65 | 3 | 35 |
| AcrIIC3-AcrIIC2-Aca3\|WP_042743680.1 | 69 | 14 | 44 | 4.30E-06 | HTH_31 | 64 | 6 | 36 |
| AcrIIC3-AcrIIC2-Aca3\|WP_042743680.1 | 69 | 14 | 44 | 1.30E-05 | HTH_23 | 50 | 11 | 39 |
| AcrIIC3-AcrIIC2-Aca3\|WP_042743680.1 | 69 | 14 | 39 | 6.50E-05 | HTH_24 | 48 | 9 | 34 |
| AcrIIC4-Aca3\|WP_049372634.1 | 140 | 1 | 102 | 2.40E-16 | DUF1870 | 118 | 1 | 110 |
| AcrIIC4-Aca3\|WP_049372634.1 | 140 | 5 | 45 | 4.70E-07 | HTH_31 | 64 | 5 | 45 |
| AcrIIC4-Aca3\|WP_049372634.1 | 140 | 5 | 49 | 1.30E-06 | HTH_XRE | 56 | 1 | 42 |
| AcrIIC4-Aca3\|WP_049372634.1 | 140 | 6 | 45 | 0.00012 | HTH_19 | 65 | 4 | 43 |
| AcrIIC4-Aca3\|WP_049372634.1 | 140 | 6 | 45 | 0.00013 | HTH_3 | 55 | 1 | 39 |
| AcrIIC5-Aca3\|WP_002642160.1 | 142 | 1 | 107 | 1.40E-14 | DUF1870 | 118 | 1 | 114 |
| AcrIIC5-Aca3\|WP_002642160.1 | 142 | 5 | 50 | 0.00057 | HTH_XRE | 56 | 1 | 45 |
| AcrIIC5-Aca3\|WP_002642160.1 | 142 | 5 | 48 | 0.00059 | HTH_31 | 64 | 5 | 48 |

**Table S8: The nine Aca families, their best Pfam HTH match, and co-localized Acr families**

| Aca family | HTH domains | Associated Acr families |
| --- | --- | --- |
| *Aca1* | *HTH_8, HTH_XRE, HTH_31* | *AcrIF, AcrIE* |
| *Aca2* | *DUF1870* | *AcrIF, AcrIIC* |
| *Aca3* | *HTH_19, HTH_XRE* | *AcrIIC* |
| *Aca4* | *HTH_23* | *AcrIF* |
| *Aca5* | *HTH_28, HTH_3* | *AcrIF* |
| *Aca6* | *HTH_3* | *AcrIF* |
| *Aca7* | *HTH_3* | *AcrIF* |
| *Aca8* | *HTH_23* | *AcrID* |
| *AcrIIA1* | *HTH_26, HTH_3* | *AcrIIA* |
